# Supplementary material for: Androgen receptor signalling in macrophages promotes TREM-1-mediated prostate cancer cell line migration and invasion
Source: Nat Commun. 2020 Sep 9;11:4498. doi: 10.1038/s41467-020-18313-y (PMC7481219; doi:10.1038/s41467-020-18313-y)
Supplement: Supplementary file 1 — Supplementary Information [file 41467_2020_18313_MOESM1_ESM.pdf]

Supplementary information

Androgen Receptor Signalling in Macrophages Promotes TREM-1 mediated Prostate Cancer Cell Line Migration and Invasion

Cioni B, Zaalberg A et al.,

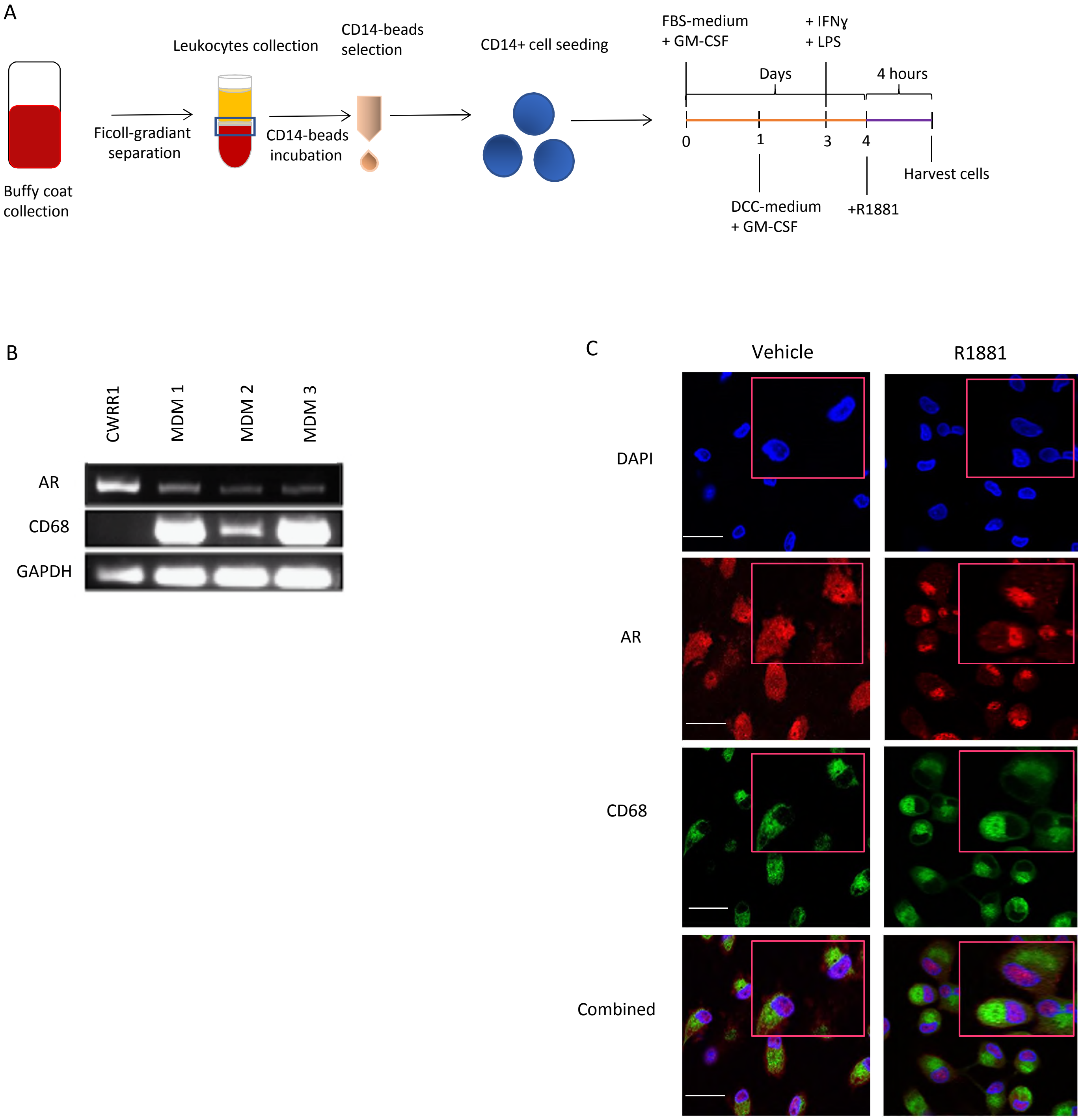

Supplementary Figure 1. AR expression and nuclear translocation in monocyte-derived macrophages. A) Workflow showing the generation of MDMs isolated from peripheral blood buffy coats of healthy donors. B) RT-PCR showing *AR* expression in MDM cells isolated from the peripheral blood of three donors (MDM1-3). *CD68* expression was included as activated pan-macrophage marker, *GAPDH* expression was used as a loading control. C) MDMs were stimulated with vehicle or R1881 to assess the nuclear translocation of AR upon R1881 stimulation. Immunofluorescence staining for AR (red) and CD68 (green). DAPI (blue) stains the DNA.. Scale bar = 50 µm (inserts: Scale bar = 100 µm*)*.

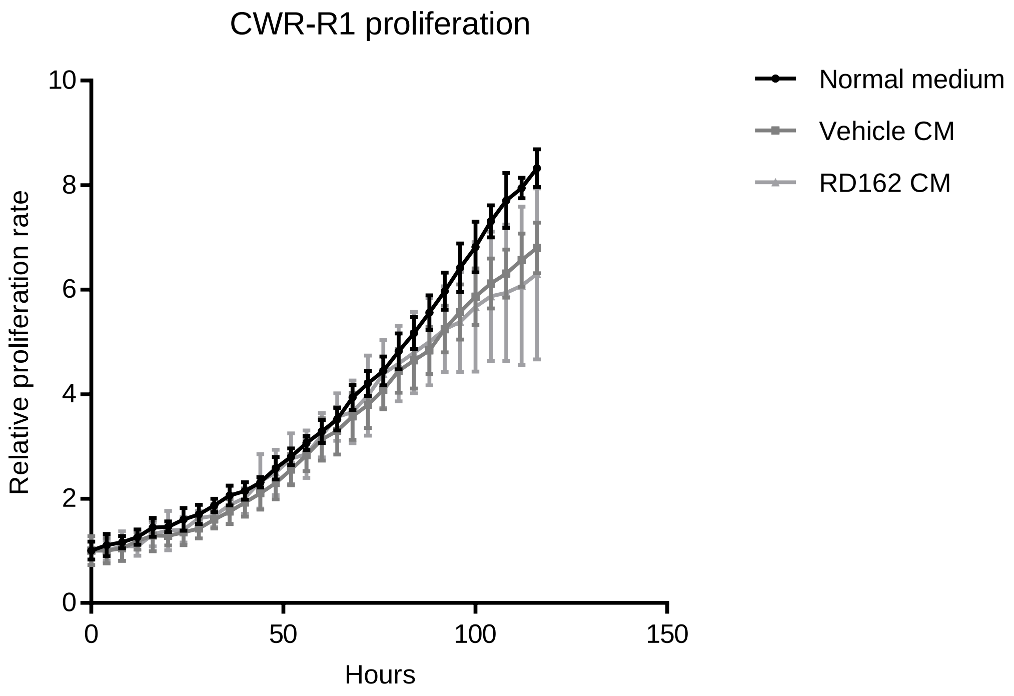

Supplementary Figure 2. Proliferation assay of CWR-R1 cells cultured in THP-1 conditioned medium. Proliferation of CWR-R1 cells cultured in normal medium (NM) alone or in combination with conditioned medium of THP-1$^{PMA;IFNG;LPS}$ cells exposed to vehicle (NM + Vehicle CM.) or RD162 (NM + RD162 CM). Error bars show the s.d. of three independent experiments. Source data are provided as a source datafile.

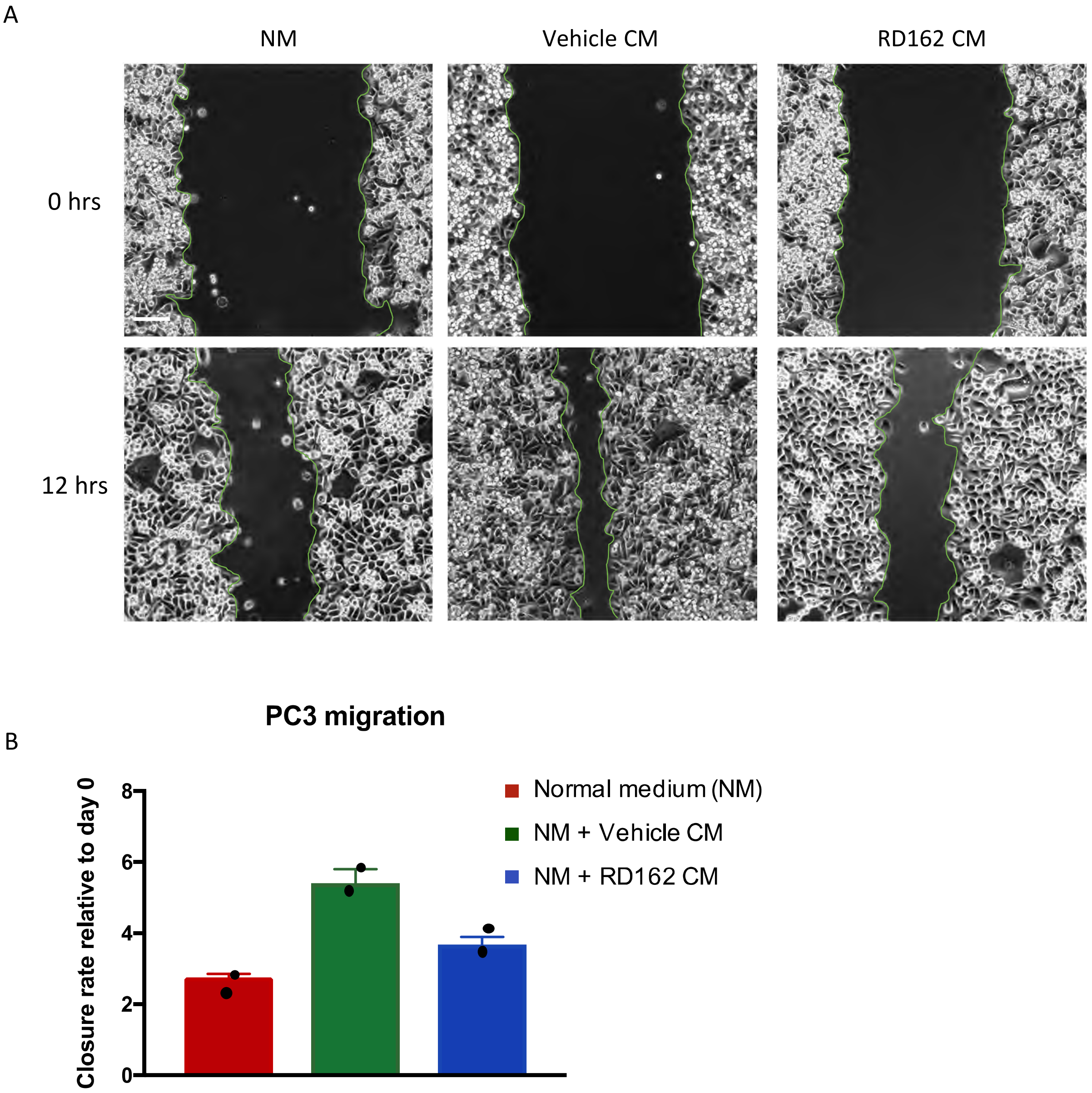

Supplementary Figure 3. AR signalling in THP-1 cells affects AR-negative PC3 PCa cells migration. A) Scratch assay of AR-negative human PC3 PCa cell line cultured in normal medium (NM) alone or in combination with conditioned medium (CM) of THP-1$^{PMA;IFNG;LPS}$ cells exposed to vehicle or RD162 for 0 hrs and 12 hrs. Scale bar = 200 µm B) Quantification of 2 independent scratch assays with three technical replicates in each experiment. PC3 cell migration is assessed through closure of the scratch after 12 hrs relative to day 0. Data points show mean values and error bars the s.e.m. Source data are provided as a source datafile.

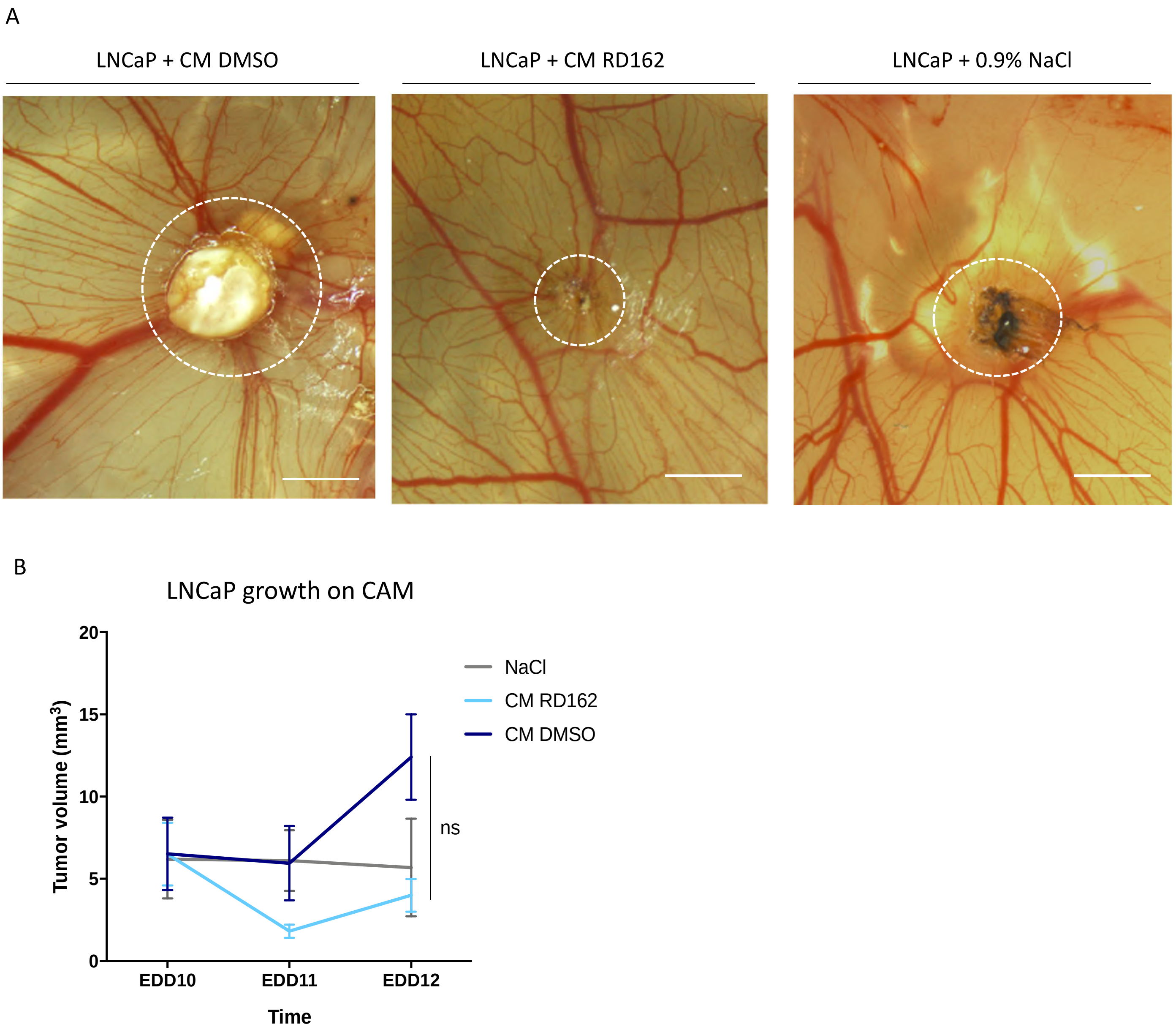

Supplementary Figure 4. Chick chorioallantoic membrane (CAM) tumour grafts from LNCaP cells. A) Representative images of LNCaP-derived tumours growing on the CAMs treated with conditioned medium of NaCl, RD162 or DMSO treated THP-1$^{PMA;IFNG;LPS}$ cells. Scale bar = 500 µm. B) Growth curves of LNCaP tumours grafted into CAMs showing tumour volume in mm^3 at different time points. Error bars represent s.e.m. of 1-7 biological replicates per condition in one experiment with one batch of THP-1$^{PMA;IFNG;LPS}$ cells CM (DMSO CM and RD162 CM) conditioned medium. p=0.28. p-value comparing DMSO CM versus RD162 CM was calculated using a Two-way Anova test with a cut-off for significance of 0.05. NS: no statistically significant difference. Source data are provided as a source datafile.

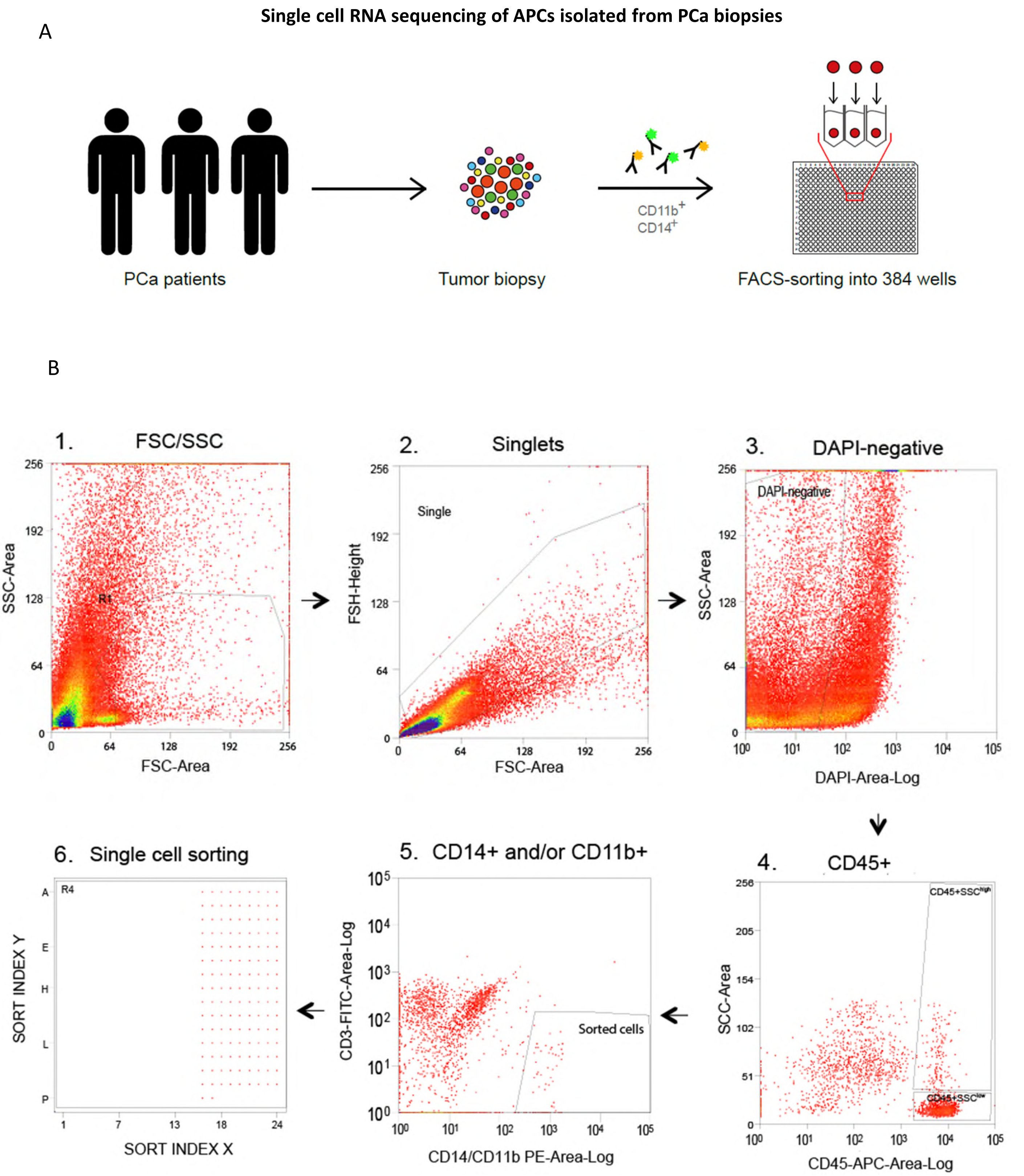

Supplementary Figure 5. Gating strategy for macrophage single cell sorting. A) Prostate biopsies were collected from 3 patients and processed for single cell suspension. CD14+ and/or CD11b+ single cells were sorted into 384 well plates and processed further for single cell RNA sequencing. After filtering 891 cells were evaluable. B) Macrophage like cells were first selected based on FSC-SSC (panel 1), followed by doublets exclusion (panel 2) and DAPI-negative selection (panel 3). CD45- cells were then excluded (panel 4), while CD45+ cells with low SCC scatter were excluded as possible lymphocytes (panel 4). Subsequently, CD45+ and/or CD11b+ cells were selected and CD3+ cells were excluded (panel 5). CD14+ and/or CD11b+ macrophages were isolated in 384 well plates (panel 6). A total of 2688 cells from 3 patients were then processed and submitted for single cell RNA sequencing.

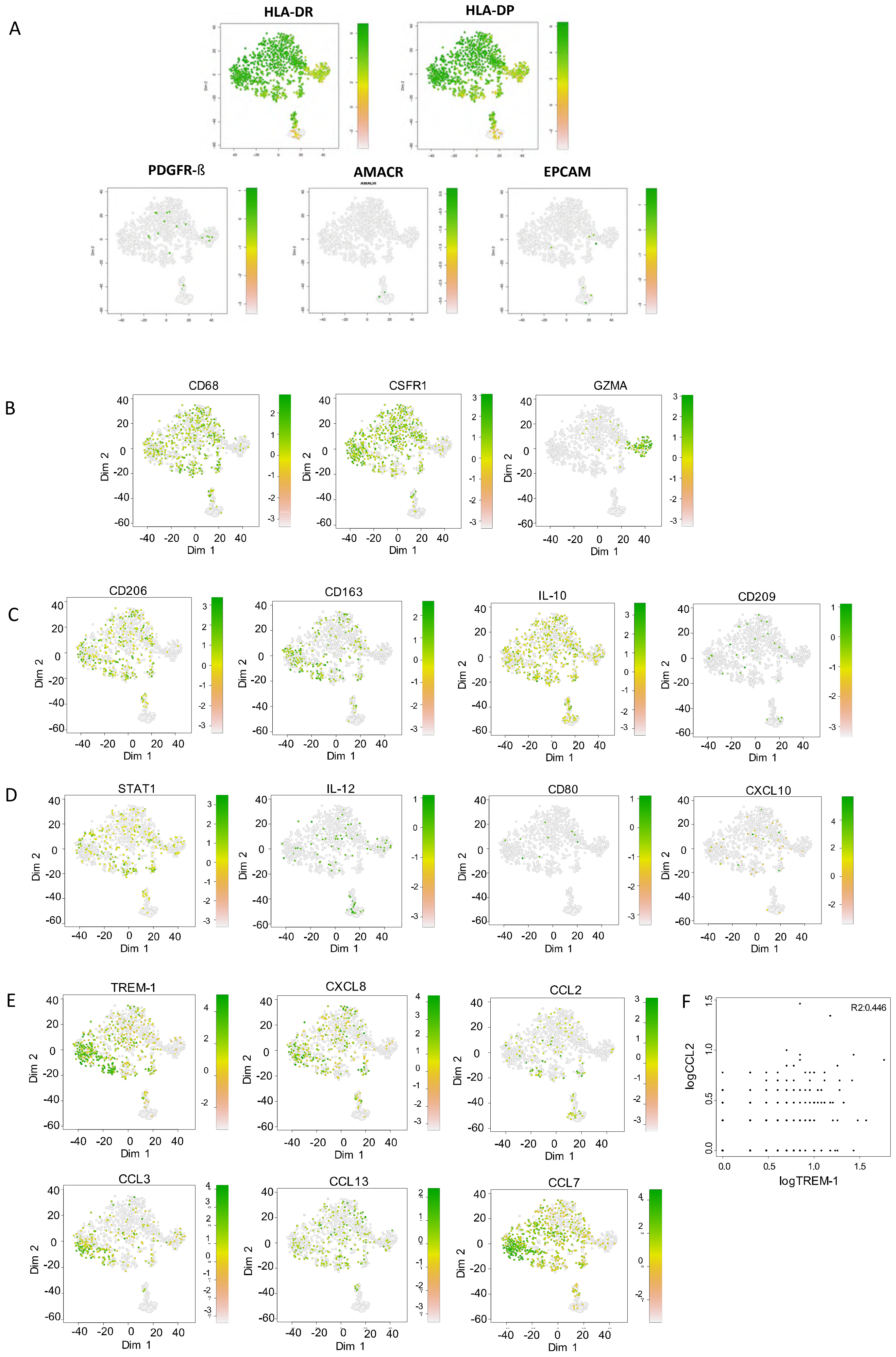

Supplementary Figure 6. Expression of macrophage markers and chemokines in CD14+ and/or CD11b+ cells isolated from PCa biopsies A) In the tSNE plots, expression of the antigen presenting cells markers *HLA-DR* and *HLA-DP*, the mesenchymal marker *PDGFR-β* and the epithelial markers *AMACR* and *EPCAM* is indicated. B) tSNE plots showing the expression levels of macrophage markers (*CD68, CSFR1* and *GZMA*) and C) the expression levels of M2-like markers *CD206*, CD163, *IL-10* and *CD209* D) the expression levels of M1-like markers *STAT1*, *IL-12*, *CD80* and *CXCL10* E) TREM-1 and its associated cytokines *CXCL8, CCL2, CCL3, CCL13* and *CCL7* F) Correlation plot between CCL2 and TREM-1 expression. Pearson R2 is shown. Every dot represents a single cell whereas colour reflects RNA level of the indicated gene proportional to the log scale shown on the right. Light grey dots represent no detectable expression of the specific gene in the single cell.

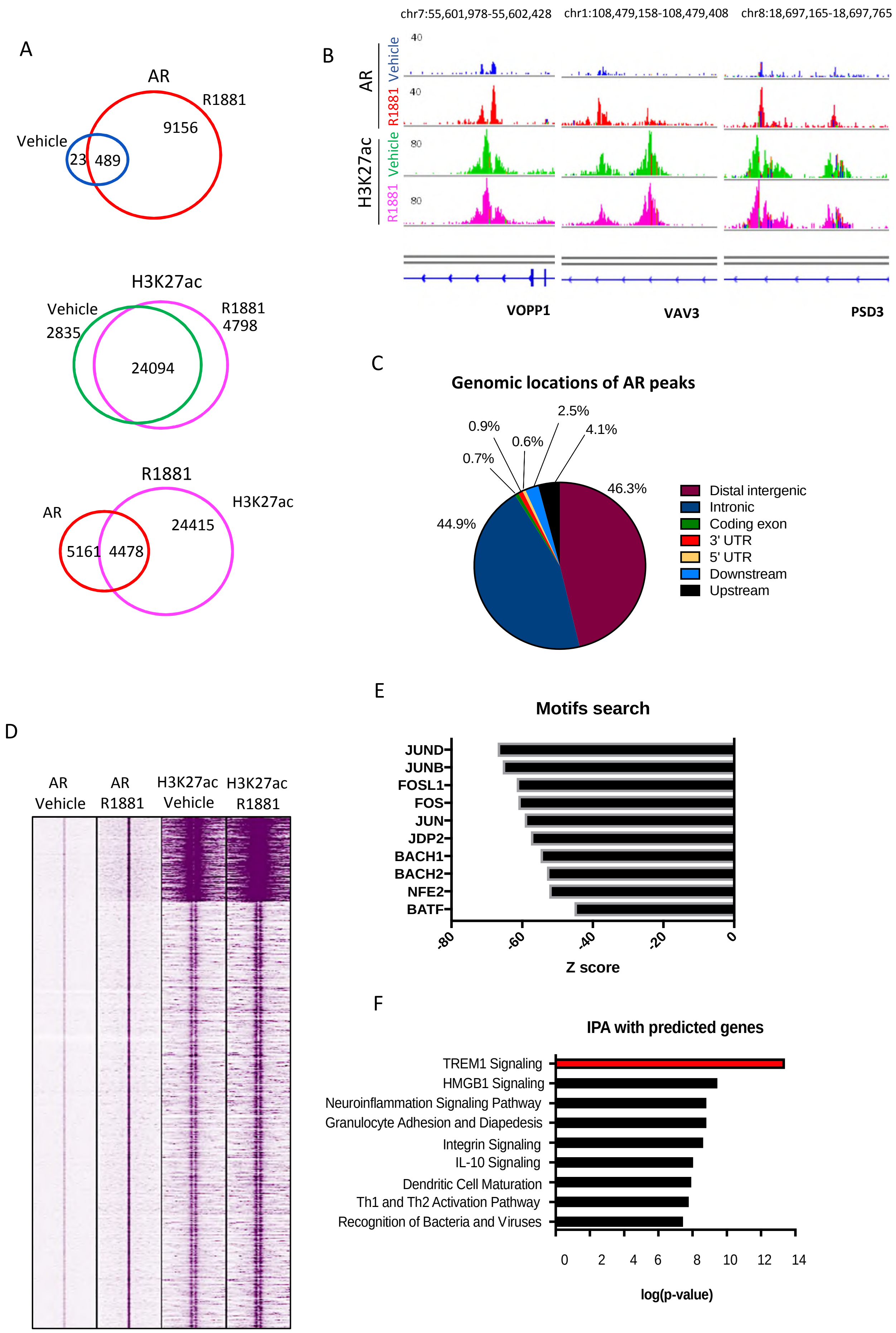

Supplementary Figure 7. AR and H3K27ac ChIP-sequencing in MDMs. A) Venn diagram shows the level of overlap between AR peaks in vehicle and R1881 conditions (top), the overlap between H3K27ac peaks in vehicle and R1881 (middle), and the overlap between AR and H3K27ac peaks (bottom). B) Genome browser snapshot of AR and H3K27ac peaks in MDM cells. Genomic coordinates, gene names and tag counts are indicated. AR binding sites in vehicle and R1881 stimulated conditions are depicted in blue and red, respectively. H3K27ac binding sites in vehicle and R1881 stimulated conditions are depicted in green and purple, respectively. Range of normalized read counts are shown on the y axis. C) Genomic distribution of AR binding sites relative to the most proximal gene. D) Clustered heatmap depicts all AR binding sites vertically aligned in MDM cells, within a 5kb window. H3K27ac peaks are shown for the same genomic locations. E) Motif analysis of AR sites identifies members of the AP-1 complex, including FOS and JUN as most enriched. Z-score of enrichment is shown on the x axis. F) Ingenuity pathway analysis of genes most proximal to AR binding sites in MDM cells identifies TREM-1 as the most enriched signalling pathway (red).

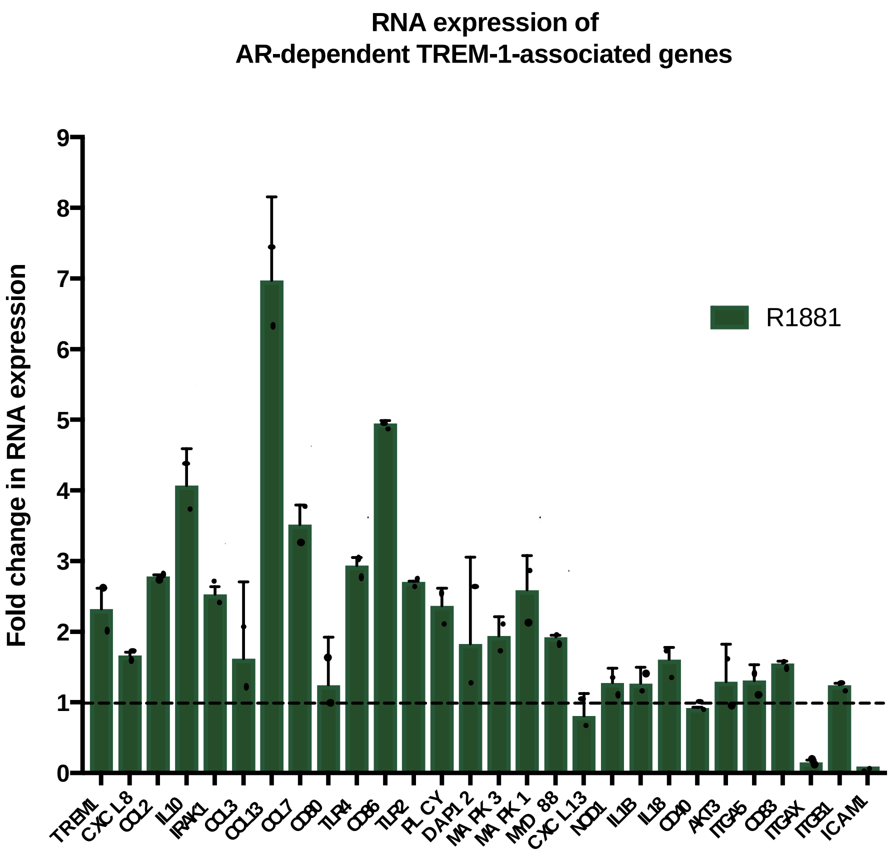

Supplementary Figure 8. Expression of TREM-1 associated genes in vehicle and R1881 exposed THP-1 cells. Fold change RNA expression levels of AR-dependent TREM-1 associated genes relative to *TBP* expression and normalized to vehicle conditions in THP-1$^{PMA;IFNG;LPS}$ cells (dotted line). Data points show mean values and error bars the s.e.m. of two independent experiments with three technical replicates each. Source data are provided as a source datafile

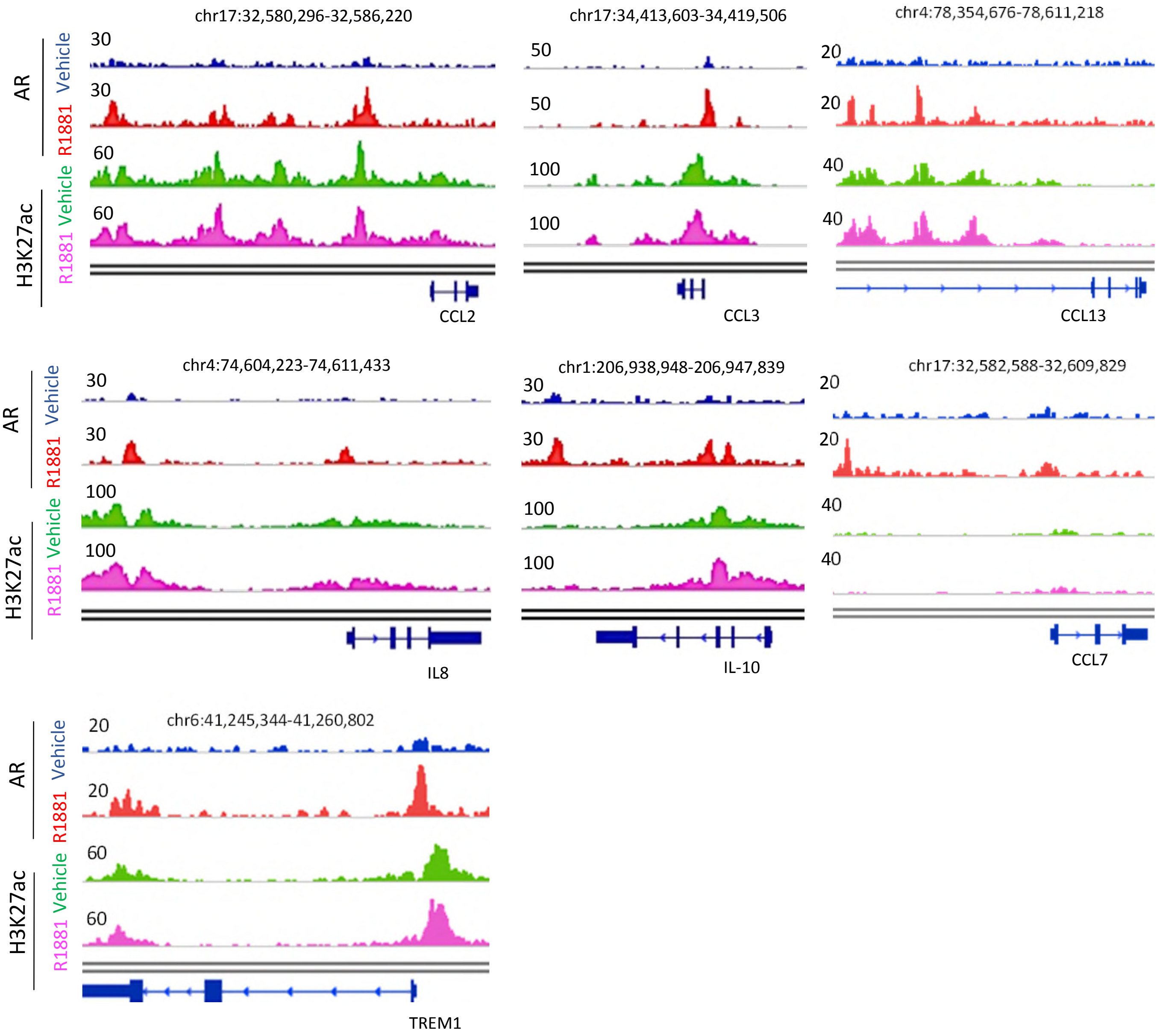

Supplementary Figure 9. Genome browser snapshot of AR and H3K27ac peaks in TREM-1 associated genes in THP-1 cells. Genomic coordinates, gene names and tag counts are indicated. AR ChIP-seq data in vehicle and R1881 treated THP-$1^{PMA;IFNG;LPS}$ cells are depicted in blue and red, respectively. H3K27ac binding sites in vehicle and R1881 treated THP-$1^{PMA;IFNG;LPS}$ cells are indicated in green and purple, respectively. Range of normalized read counts are shown on the y-axis.

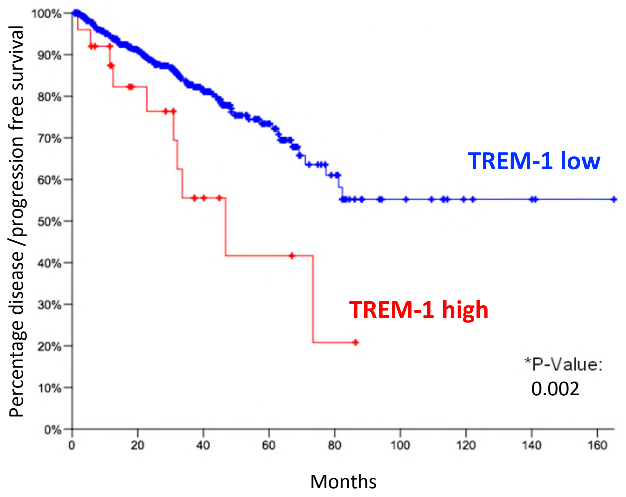

Supplementary Figure 10. Kaplan-Meier analysis of disease/progression-free survival in PCa TCGA database related to *TREM-1* expression. Kaplan-Meier curves of disease/progression-free survival in PCa patients in relation to levels of *TREM-1* expression (TCGA database, number of patients = 491), here with a more stringent Z-score of ± 2. Twenty-six cases with a high *TREM-1* expression (red line; 10 relapses/disease progressions) and 465 cases with a low *TREM-1* expression (blue line; 81 relapses/disease progressions). P=0.002. Log-rank chi square test was used to calculate the p value with a cut-off for significance of 0.05. Source data are provided as a source datafile.

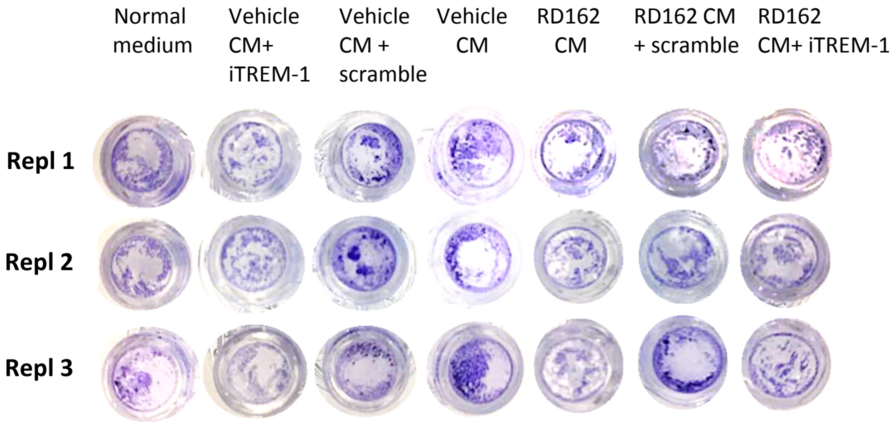

Supplementary Figure 11. Transwell invasion assay of PCa cells cultured in conditioned medium of TREM-1 blocked THP-1 cells. Representative transwell plate of an invasion assay of CWR-R1 cells cultured in normal medium alone or in a combination of normal medium and CM of THP-1$^{PMA;IFNG;LPS}$ cells stimulated with vehicle and TREM-1 inhibitory peptide, vehicle with scramble peptide, vehicle alone, RD162, RD162 with scramble peptide or RD162 and TREM-1 inhibitory peptide. Cells that invade the matrigel and passed through the membrane after 72 hrs of culture were stained with crystal violet.

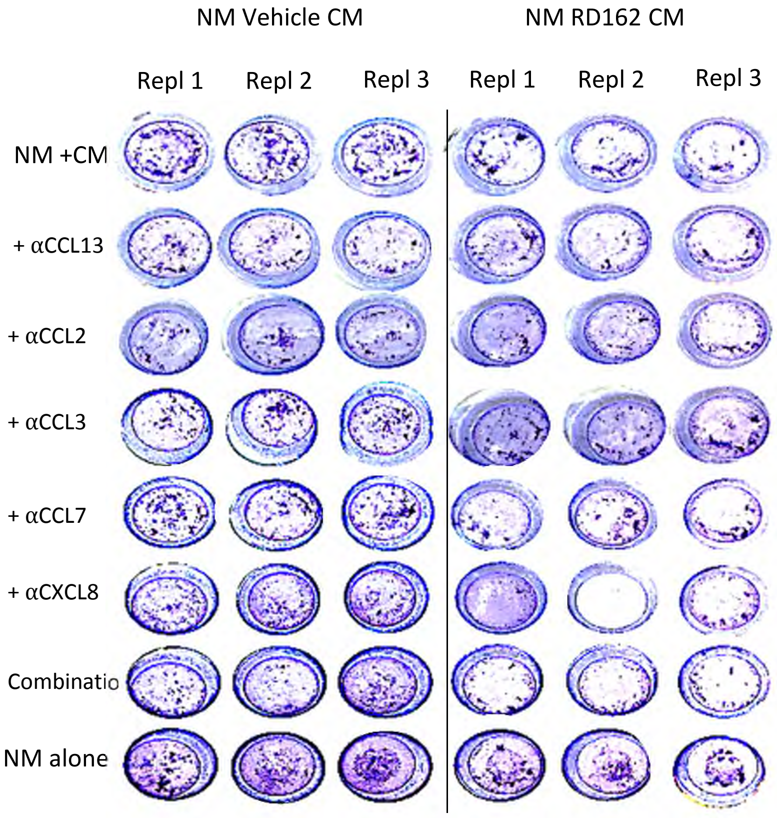

Supplementary Figure 12. Transwell migration assay of PCa cells cultured in conditioned medium of THP-1 cells and blocking antibodies against cytokines. Representative transwell migration assay of CWR-R1 cells cultured in normal medium (NM) in combination with conditioned medium (CM) of THP-1$^{PMA;IFNG;LPS}$ cells stimulated with vehicle (left panel) or RD162 (right panel). NM in combination with CM of THP-1$^{PMA;IFNG;LPS}$ cells was supplemented with no blocking antibodies (row 1), blocking antibodies against CCL13, CCL2, CCL3, CCL7, CXCL8 or the combination of all (rows 2-7). Migration of CWR-R1 cells cultured in NM only is shown in row 8. Cells that passed through the membrane after 72 hrs of culture were stained with crystal violet.

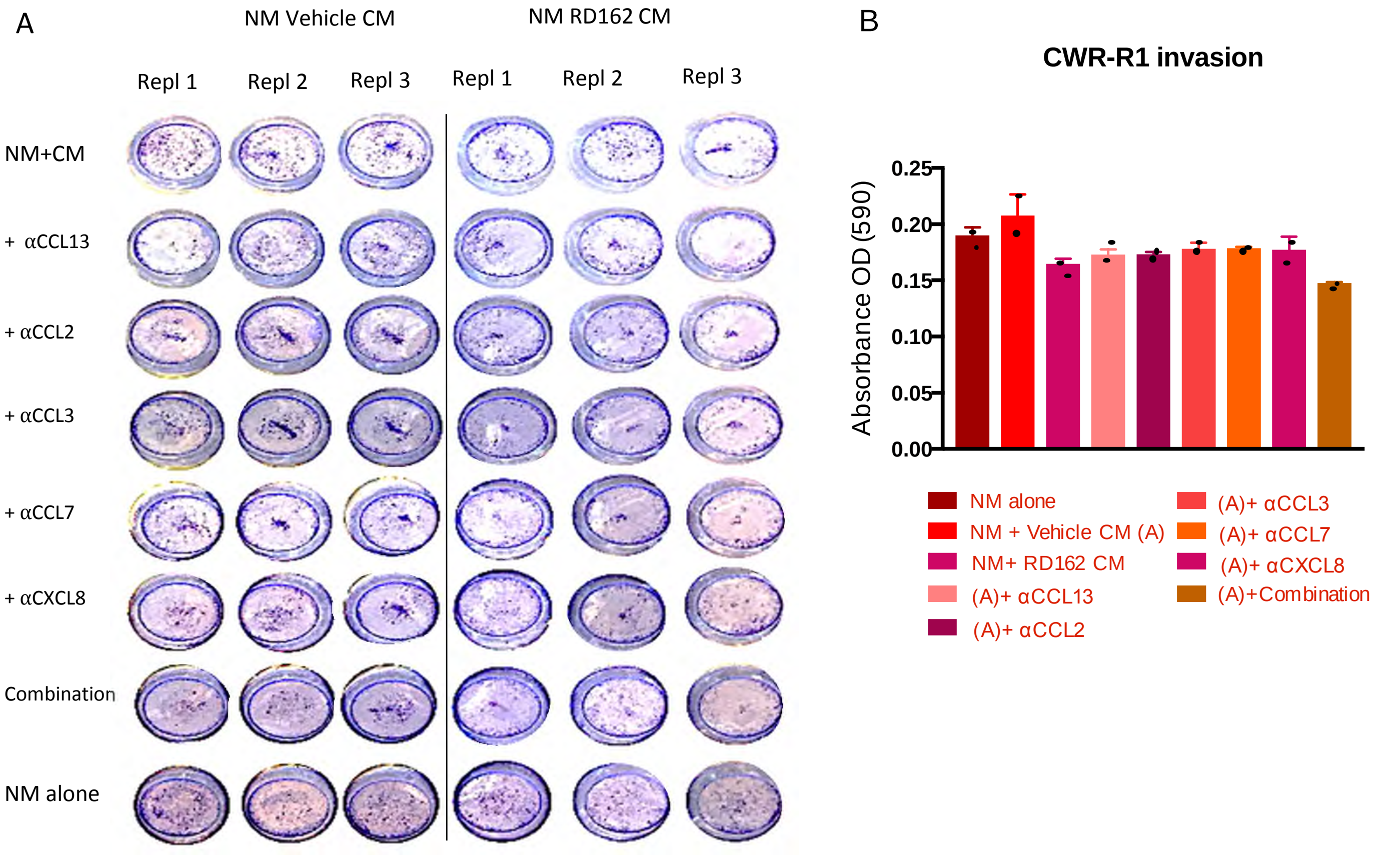

Supplementary Figure 13. Transwell invasion assays of PCa cells cultured in THP-1 cells conditioned medium and blocking antibodies against CCL2, CXCL8, CCL3, CCL13 and CCL7. A) Representative transwell invasion assay of CWR-R1 cells cultured in normal medium (NM) in combination with conditioned medium (CM) of THP-1$^{PMA;IFNG;LPS}$ cells stimulated with vehicle (left panel) or RD162 (right panel). NM in combination with CM of THP-1$^{PMA;IFNG;LPS}$ cells was supplemented with no blocking antibodies (row 1), blocking antibodies against CCL13, CCL2, CCL3, CCL7, CXCL8 or the combination of all (rows 2-7). Migration of CWR-R1 cells cultured in NM only is shown in row 8. Cells that passed through the membrane after 72 hrs of culture were stained with crystal violet.

B) Quantification of the transwell invasion assays. Data points show mean values and error bars the s.e.m. of two independent experiments with three technical replicates each. Source data are provided as a source datafile.

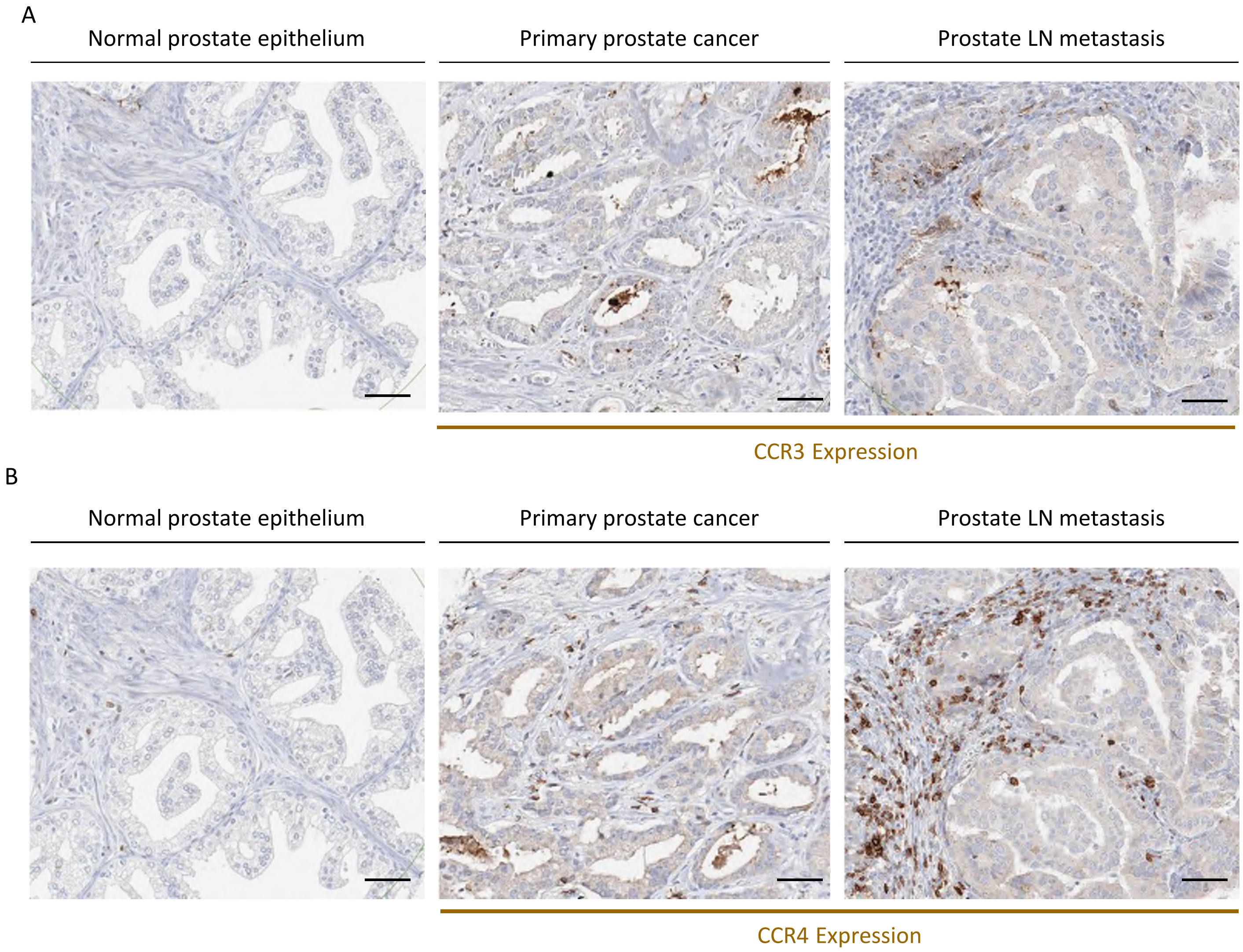

Supplementary Figure 14. Expression of CCR3 and CCR4 by immunohistochemistry. Representative images showing the expression of chemokine receptor 3 (CCR3) and CCR4 in human normal prostate and prostate cancer tissue as well as lymph node metastases. Scale bar = 50 µm

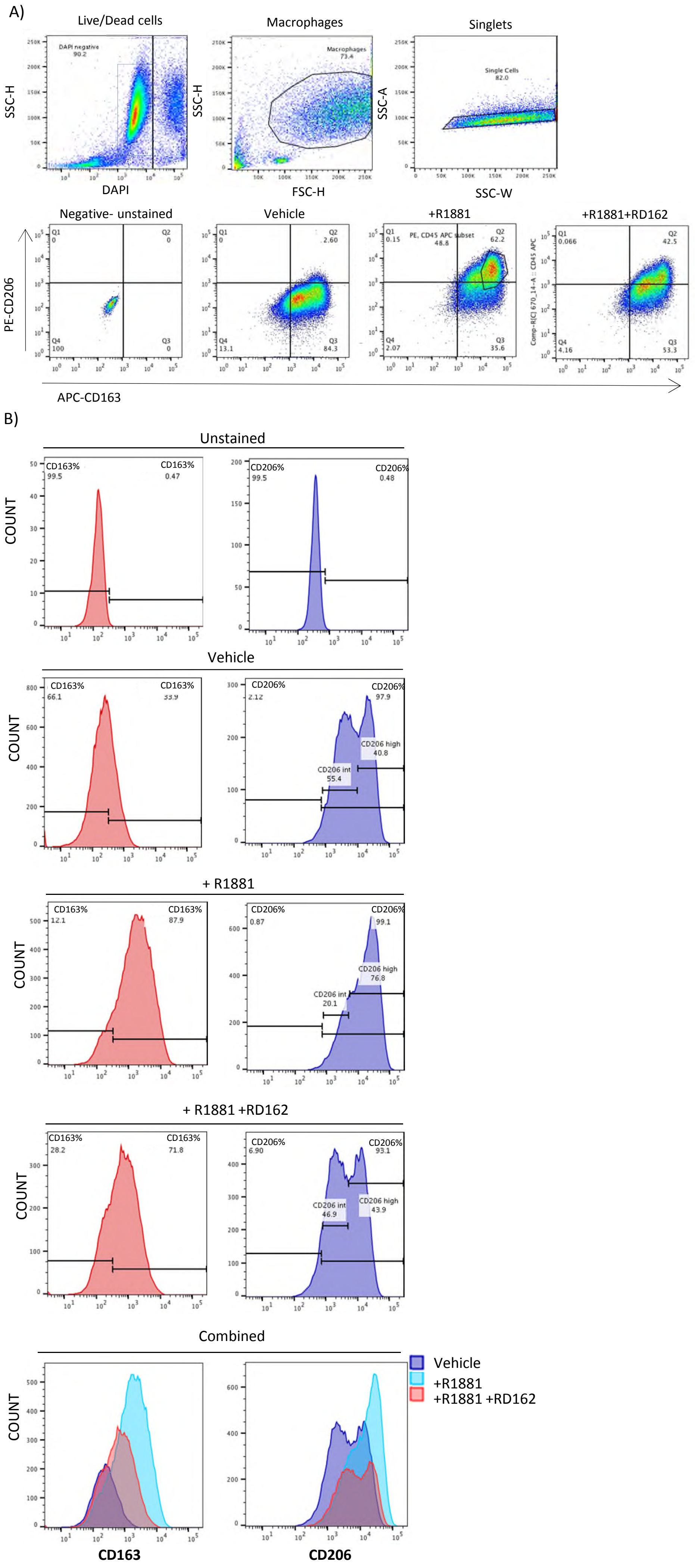

Supplementary Figure 15. Flow cytometry analysis of CD163 and CD206 markers in testosterone-stimulated MDM cells. A) Gating strategy used to identify alive cells (DAPI negative), macrophage like cells based on FSC-SSC (SSC-C/FSC-H) and single cells (SSC-A/SSC-W)(upper panel). MDM cells were exposed to vehicle, R1881 or R1881 in combination with RD162 for 24 hrs. APC and PE channels were used to identify CD163 and CD206 expression in these cells, respectively (lower panel). B) Cell count and relative percentage of CD163+ (left panel) and CD206+ (right panel) MDM cells stimulated with vehicle, R1881 alone or in combination with RD162. Lower row shows all three conditions combined. Unstained sample was used as negative control.

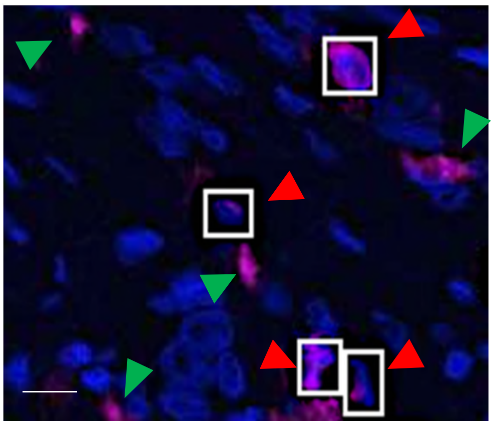

Supplementary Figure 16. Identification of CD163 true positive and false positive cells in PCa biopsies. CD163 true positive (red) and false positive (green) macrophages were identified in PCa biopsies based on signal threshold and localization. Scale bar = 100 μm

Figure 2C

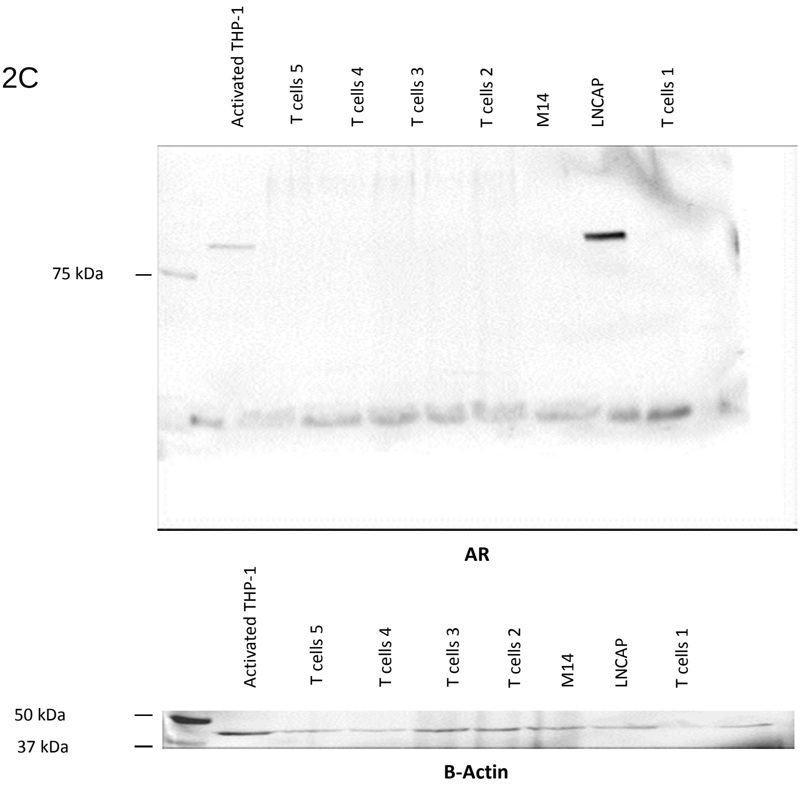

Figure 2D

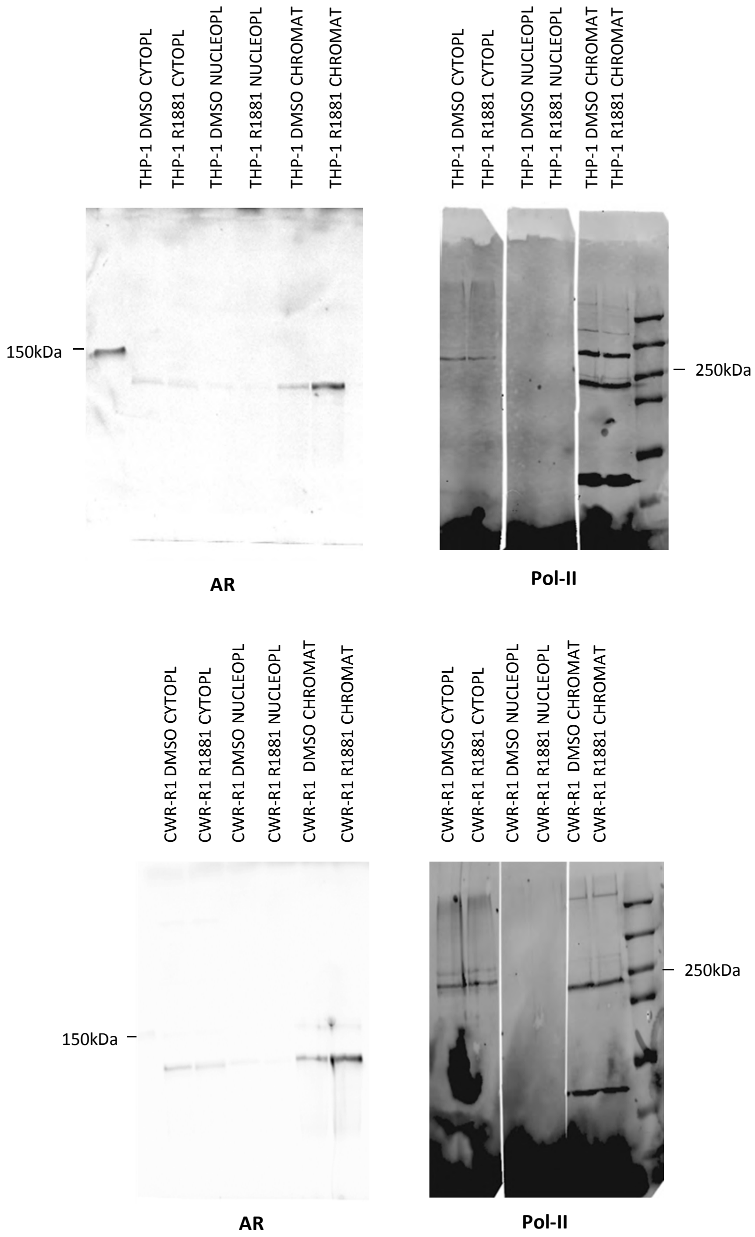

Supplementary Figure 17. Full blots of Figures 2C and 2D

Symbol	Gene Name	Gene Symbol	Expected	Type(s)	Gene ID for Human
TYROBP	TYRO protein tyrosine kinase binding protein	TYROBP	Up	Transmembrane receptor	7305
TREM1	Triggering receptor expressed on myeloid cells 1	TREM1	Up	Transmembrane receptor	54210
TNF	Tumor necrosis factor	TNF	Up	Cytokine	7124
TLR9	Toll like receptor 9	TLR9	Up	Transmembrane receptor	54106
TLR8	Toll like receptor 8	TLR8	Up	Transmembrane receptor	51311
TLR6	Toll like receptor 6	TLR6	Up	Transmembrane receptor	10333
TLR4	Toll like receptor 4	TLR4	Up	Transmembrane receptor	7099
TLR2	Toll like receptor 2	TLR2	Up	Transmembrane receptor	7097
TRL1	Toll like receptor 1	TLR1	Up	Transmembrane receptor	7096
STAT5B	Signal transducer and activator of transcription 5B	STAT5B	Up	Transcription regulator	6777
NOD2	Nucleotide binding oligomerization domain containing 2	NOD2	Up	Other	64127
NOD1	Nucleotide binding oligomerization domain containing 1	NOD1	Up	Other	10392
NLRP12	NLR family pyrin domain containing 12	NLRP12	Up	Other	91662
NLRP6	NLR family pyrin domain containing 6	NLRP6	Up	G-protein coupled receptor	171389
NLRP3	NLR family pyrin domain containing 3	NLRP3	Up	Other	197358
NLRP2	NLR family pyrin domain containing 2	NLRP2	Up	Other	55655
NLRC5	NLR family CARD domain containing 5	NLRC5	Up	Transcription regulator	84166
NLRC3	NLR family CARD domain containing 3	NLRC3	Up	Other	197358
MYD88	Myeloid differentiation primary response 88	MYD88	Up	Other	4615
MAPK1	Mitogen-activated protein kinase 1	MAPK1	Up	Kinase	5594
JAK2	Janus kinase 2	JAK2	Up	Kinase	3717
ITGB1	Integrin subunit beta 1	ITGB1	Up	Transmembrane receptor	3688
ITGAX	Integrin subunit alpha X	ITGAX	Up	Transmembrane receptor	3687
ITGA5	Integrin subunit alpha 5	ITGA5	Up	Transmembrane receptor	3678
IL1B	Interleukin 1 beta	IL1B	Up	Cytokine	3553
IL18	Interleukin 18	IL18	Up	Cytokine	3606
IL10	Interleukin 10	IL10	Up	Cytokine	3586
ICAM1	Intercellular adhesion molecule 1	ICAM1	Up	Transmembrane receptor	3383
CXCL8	C-X-C motif chemokine ligand 8	CXCL8	Up	Cytokine	3576
CCL3	C-C motif chemokine ligand 3	CXCL3	Up	Cytokine	2921
CSF2	Colony stimulating factor 2	CSF2	Up	Cytokine	1437
CIITA	Class II major histocompatability complex transactivator	CIITA	Up	Transcription regulator	4261
CD86	CD86 molecule	CD86	Up	Transmembrane receptor	942
CD83	CD83 molecule	CD83	Up	Transmembrane receptor	9308
CD40	CD40 molecule	CD40	Up	Transmembrane receptor	958
CCL7	C-C motif chemokine ligand 7	CCL7	Up	Cytokine	6354
CCL2	C-C motif chemokine 2	CCL2	Up	Cytokine	6347
AKT3	AKT serine/threonine kinase 3	AKT3	Up	Kinase	10000

Supplementary Table 1. Genes predicted to be AR-regulated and found in the TREM-1 signalling pathway.

Matched patients	Treatment	Gleason	pT	PSA	Age	Dosage	Length of the treatment (weeks)
1	yes	7	2	31-50	61-70	150mg	20
1	no	7	2	31-50	61-70	-	-
2	yes	7	2	0-10	51-60	50mg	21
2	no	7	2	0-10	51-60	-	-
3	yes	8	2	11-20	61-70	50mg	18
3	no	8	2	11-20	61-70	-	-
4	yes	8	3	0-10	61-70	150mg	13
4	no	8	3	0-10	61-70	-	-
5	yes	6	3	21-30	61-70	150mg	12
5	no	6	3	21-30	61-70	-	-
6	yes	6	3	11-20	51-60	150mg	12
6	no	6	3	11-20	51-60	-	-
7	yes	6	2	0-10	61-70	150mg	39
7	no	6	2	0-10	61-70	-	-
8	yes	6	2	0-10	61-70	150mg	15
8	no	6	2	11-20	51-60	-	-
9	yes	6	2	11-20	51-60	150mg	16
9	no	7	3	11-20	51-60	-	-
10	yes	7	3	11-20	51-60	150mg	12
10	no	8	2	11-20	61-70	-	-

Supplementary Table 2. Clinico-pathological characteristics of PCa patients included in this study. Patients were treated with bicalutamide for 9-39 weeks in doses between 50 and 150 mg daily Patients were matched (1-10) based on bicalutamide treatment (yes/no), Gleason score (6-8), pT classification (2-3), initial PSA levels (0-50) and age (51-70).

		Group 1	Group 2
**Expected**	Untreated (Control)	10	0
	Treated (Cases)	0	10
**Observed**	Untreated (Control)	7	3
	Treated (Cases)	2	8

p=0.024

Supplementary Table 3. Expected and observed clustering of untreated and treated patients. Expected clustering was based on clinical records (treated and not-treated), while observed clustering was based on the different CD163+ populations in the two groups. P=0.024. Pearson correlation test was used to calculate the p value with a cut-off for significance of 0.05.

Treatment	CD163+	CD163-	Group
yes	1321	4954	1
no	2090	6487	1
yes	1830	5034	2
no	4224	9869	2
yes	3595	11647	3
no	122	333	3
yes	8669	39340	4
no	3970	10420	4
yes	16222	50177	5
no	489	2607	5
yes	2778	29941	6
no	376	755	6
yes	5976	28131	7
no	754	4577	7
yes	17532	46047	8
no	1779	9928	8
yes	819	4666	9
no	21264	93620	9
yes	3859	13676	10
no	7520	17405	10
	**P value CTRL Vs Cases**	**P value CTRL Vs Cases**	
	0.538	0.555	

Supplementary Table 4. Quantification of CD163+ and CD163- cells in untreated and treated patients. Absolute numbers of CD163+ (HLA-DRA+ and/or CD14+) and CD163- (HLA-DRA+ and/or CD14+) cells are shown in the table. Groups are explained in Supplementary Table 2.

Primers used for PCR and qPCR (Invitrogen)		
**Gene**	**Forward**	**Reverse**
**AKT3**	GCAGAGGCAAGAAGAGGAGA	ACT TGC CTT CTC TCG AAC CA
**AR**	GACACCGACACTGCCTTAC	TAGGGCTGGGAAGG-GTCTAC
**CCL13**	ATCTCCTTGCAGAGGCTGAA	AGAAGAGGAGGCCAGAGGAG
**CCL2**	CCCCAGTCACCTGCTGTTAT	TCCTGAACCCACTTCTGCTT
**CCL2**	CCCCAGTCACCTGCTGTTAT	TGGAATCCTGAACCCACTTC
**CCL3**	TTC CGT CAC CTG CTC AGA AT	TGG CTG CTC GTC TCA AAG TA
**CCL3**	TGCAACCAGTTCTCTGCATC	ACCTGGAGACTAGGGGGCTA
**CCL7**	AAGA=CAGAGGCTGGAGAGCTA	GGTTTTCTTGTCCAGGTGCT
**CCL7**	ATGAAAGCCTCTGCAGCACT	GGACAGTGGCTACTGGTGGT
**CD163**	GAAGGCTCTGGACCAATCTG	GCTCAGATCTGCTCCCTTTG
**CD209**	TGCTGCTGTCCTCATTTTTG	ACTTGCAGTGCCTCCTCAGT
**CD40**	GCA GGC ACA AAC AAG ACT GA	TCG GGA AAA TTG ATC TCC TG
**CD68**	AGGCTGGCTGTGCTTTT	CTTCCCTG-GACCTTGGTT
**CD83**	GGA TGA GAG GGT GCT ATC CA	CTT CGT GAA GTC CCT TCT GC
**CXCL8**	CCA GCC TCC TCA GAA ACA GA	TCC CTC CAG CAG TGA AGA AG
**CXCL8**	TAGCAAAATTGAGGCCAAGG	GGACTTGTGGATCCTGGCTA
**hCycloA**	AGCATGTGGTGTTTGGCAAA	TCGAGTTGTCCACAGTCAGC
**DAP12**	TGATTGCAGTTGCTCTACGG	TAAGGCGACTCGGTCTCAGT
**GAPDH**	ACCCAGAAGACTGTGGATGG	TTCTAGACGGCAGGTCAGGT
**hVimentin**	GCGAGGAGAGCAGGATTTCTC	ACCAGAGGGAGTGAATCCAGA
**ICAM1**	GGC TGG AGC TGT TTG AGA AC	ACT GTG GGG TTC AAC CTC TG
**IL-10**	TGAAGGATCAGCTGGACAAC	GCCTTGATGTCTGGGTCTTG
**IL18**	AGT GGC TCA CGC CTG TAA TC	TCT ACC TCC GGA GTG CAA GT
**IL1B**	GCT GAG GAA GAT GCT GGT TC	TCC ATA TCC TGT CCC TGG AG
**ITGA5**	GTG GGC CAA CAA AGA ACA CT	TGG AGC AGG CCC AAA TAT AG
**ITGAX**	GTG GTG GTG TGA TGC TGT TC	ATA CTG CAG CCT GGA GGA GA
**ITGB1**	CAT CTG CGA GTG TGG TGT CT	GGG GTA ATT TGT CCC GAC TT
**MAPK1**	CTCCTGTGGTGCAGATGAGA	CAGCACAAGAAAAGGCAACA
**MAPK3**	ACAGTCTCTGCCCTCCAAGA	CTCATCCGTCGGGTCATAGT
**MRC1(CD206)**	GGGCAGTGAAAGCTTATGGA	CCTGTCAGGTATGTTTGCTCA
**MYD88**	GCACATGGGCACATACAGAC	GACATGGTTAGGCTCCCTCA
**NOD1**	CCA CTT CAC AGC TGG AGA CA	TGA GTG GAA GCA GCA TTT TG
**PLCgamma1**	GACATCACCTACGGGCAGTT	GGAGGAAGCTGAGCATGAAC
**TBP**	GTTCTGGGAAAATGGTGTGC	GCTGGAAAACCCAACTTCTG
**TLR2**	CTA TGA ATC AAG GCG GCC AC	AAA GAT CCT GAG CTG CCC TT
**TLR4**	CCAGCCTCCTCAGAAACAGA	TCCCTCCAGCAGTGAAGAAG
**TNF**	AGG ACC AGC TAA GAG GGA GA	CCC GGA TCA TGC TTT CAG TG
**TREM1**	AGGAGCCTCACATGCTGTTC	CACAGTTCTGGGGCTGGTAT
**TYROBP(DAP12)**	TGA TTG CAG TTG CTC TAC GG	TAA GGC GAC TCG GTC TCA GT

Supplementary Table 5. Primers used for PCR and qPCR analysis.
