# Supplementary material for: Androgen receptor signalling in macrophages promotes TREM-1-mediated prostate cancer cell line migration and invasion
Source: Nat Commun. 2020 Sep 9;11:4498. doi: 10.1038/s41467-020-18313-y (PMC7481219; doi:10.1038/s41467-020-18313-y)
Supplement: Supplementary file 3 — Reporting Summary [file 41467_2020_18313_MOESM3_ESM.pdf]

# nature research

Corresponding author(s): Bergman AM

Last updated by author(s): Jan 23, 2020

# Reporting Summary

Nature Research wishes to improve the reproducibility of the work that we publish. This form provides structure for consistency and transparency in reporting. For further information on Nature Research policies, see Authors & Referees and the Editorial Policy Checklist.

## Statistics

For all statistical analyses, confirm that the following items are present in the figure legend, table legend, main text, or Methods section.

| n/a | Confirmed | |
|---|---|---|
| ☐ | ☒ | The exact sample size ($n$) for each experimental group/condition, given as a discrete number and unit of measurement |
| ☐ | ☒ | A statement on whether measurements were taken from distinct samples or whether the same sample was measured repeatedly |
| ☐ | ☒ | The statistical test(s) used AND whether they are one- or two-sided<br>*Only common tests should be described solely by name; describe more complex techniques in the Methods section.* |
| ☐ | ☒ | A description of all covariates tested |
| ☐ | ☒ | A description of any assumptions or corrections, such as tests of normality and adjustment for multiple comparisons |
| ☐ | ☒ | A full description of the statistical parameters including central tendency (e.g. means) or other basic estimates (e.g. regression coefficient) AND variation (e.g. standard deviation) or associated estimates of uncertainty (e.g. confidence intervals) |
| ☒ | ☐ | For null hypothesis testing, the test statistic (e.g. $F$, $t$, $r$) with confidence intervals, effect sizes, degrees of freedom and $P$ value noted<br>*Give P values as exact values whenever suitable.* |
| ☒ | ☐ | For Bayesian analysis, information on the choice of priors and Markov chain Monte Carlo settings |
| ☒ | ☐ | For hierarchical and complex designs, identification of the appropriate level for tests and full reporting of outcomes |
| ☐ | ☒ | Estimates of effect sizes (e.g. Cohen's $d$, Pearson's $r$), indicating how they were calculated |

*Our web collection on statistics for biologists contains articles on many of the points above.*

## Software and code

Policy information about availability of computer code

| | |
|---|---|
| Data collection | cBioPortal for Cancer Genomic website version 1.12.1 was used in this study. dbGaP Study Accession: phs000178.v10.p8 |
| Data analysis | Software used in this study were: Rstudio software (v0.99.491 and v3.4.3), Python (v3.6.3), HALO (v2.1), ImageJ software (v1.50), Ingenuity Pathway Analysis (v2.2.1), Prism/graphPad (v7.0c), DiffBind R package (v2.4.6), Intergrative Genomics Viewer (v2.3.77(116)) |

For manuscripts utilizing custom algorithms or software that are central to the research but not yet described in published literature, software must be made available to editors/reviewers. We strongly encourage code deposition in a community repository (e.g. GitHub). See the Nature Research guidelines for submitting code & software for further information.

## Data

Policy information about availability of data

All manuscripts must include a data availability statement. This statement should provide the following information, where applicable:

- Accession codes, unique identifiers, or web links for publicly available datasets
- A list of figures that have associated raw data
- A description of any restrictions on data availability

The ChIP-seq data generated in this study have been deposited in the National Center for Biotechnology Information (NCBI) in the Gene Expression Omnibus (GEO) database under accession number GSE131381 and the single-cell RNA sequencing data under accession number GSE133094.

# Field-specific reporting

Please select the one below that is the best fit for your research. If you are not sure, read the appropriate sections before making your selection.

☒ Life sciences ☐ Behavioural & social sciences ☐ Ecological, evolutionary & environmental sciences

For a reference copy of the document with all sections, see nature.com/documents/nr-reporting-summary-flat.pdf

# Life sciences study design

All studies must disclose on these points even when the disclosure is negative.

| | |
|---|---|
| Sample size | At least three technical replicates were included in each experiment. Each experiment was performed at least 2 or 3 times. Number of patients included in this study were based on tissue availabiity. |
| Data exclusions | No data were excluded from analysis |
| Replication | Standard error of the mean was used to evaluate the reproducibility among different experiments |
| Randomization | Both cell lines and patients grouping were based on case and controls (stimulation/control and treatment/control respectively) |
| Blinding | Investigator was blind in case/control grouping during scoring of immunofluorescence staining in FFPE material from patients |

# Reporting for specific materials, systems and methods

We require information from authors about some types of materials, experimental systems and methods used in many studies. Here, indicate whether each material, system or method listed is relevant to your study. If you are not sure if a list item applies to your research, read the appropriate section before selecting a response.

## Materials & experimental systems

| n/a | Involved in the study | |
|---|---|---|
| ☐ | ☒ | Antibodies |
| ☐ | ☒ | Eukaryotic cell lines |
| ☒ | ☐ | Palaeontology |
| ☒ | ☐ | Animals and other organisms |
| ☐ | ☒ | Human research participants |
| ☒ | ☐ | Clinical data |

## Methods

| n/a | Involved in the study | |
|---|---|---|
| ☐ | ☒ | ChIP-seq |
| ☐ | ☒ | Flow cytometry |
| ☒ | ☐ | MRI-based neuroimaging |

## Antibodies

| | |
|---|---|
| Antibodies used | CCR3 clone Y31 (AbCam), CCR4 polyconal (Sigman Aldrich), anti-AR (Santa Cruz, sc-816) anti-CD68 (Dako, KP1), Alexa Fluor 488 anti-mouse (ThermoFisher Scientific), Alexa Fluor 568 anti-rabbit (ThermoFisher Scientific), anti-AMACR (clone 13H4, cat M3616, Dako, 1/1600 dilution 32 minutes at RT), anti-AR (clone SP107, cat M4074, Spring Bioscience), anti-CD14 (clone EPR3653, cat 114R-14, Cell Marque), anti-CD163 (clone 10D6, cat NCL-CD163, Leica), anti-CD20 (clone L26, cat M077, Dako), anti-HLA-DR (clone TAL1b5, cat M0746, Dako). Antibodies against CXCL8, CCL2, CCL3, CCL13 and CCL7 (all R&D Systems), anti-CD162 APC (eBioscience), anti-CD206 PE (eBioscience), anti-Pol-II (Santa Cruz sc-56767), anti-B-actin (NOvus Biological, NB600-501), anti-AR (Millipore, 06-680) and anti-H3k27ac (Active Motif, 39133). |
| Validation | Positive and negative controls were used for validation of the multiplex stainin. Validation of other antibodies was provided by the manufacturer. |

## Eukaryotic cell lines

Policy information about cell lines

| | |
|---|---|
| Cell line source(s) | THP-1 (human monocytic cell line), M14 (melanoma cell line), CWR-R1, PC3 and LNCaP (prostate cancer cell lines) were used in this study |
| Authentication | None of the cells were authenticated |
| Mycoplasma contamination | Cells were regularly tested for mycoplsma contamination and tested negative |
| Commonly misidentified lines (See ICLAC register) | No misidentified lines were used in this study |

## Human research participants

Policy information about studies involving human research participants

| | |
|---|---|
| Population characteristics | Men with prostate cancer were included in this study. Age of patients was between 50 and 70 years. Patients were untreated or treated with hormone therapy for 12-39 weeks. All patients received prostatectomy surgery. |
| Recruitment | Patients were selected based on gleason score (6-8) and previous treatment with hormone therapy |
| Ethics oversight | The study has been approved by the Institutional Review Board (IRB) from the Netherlands Cancer Institute |

Note that full information on the approval of the study protocol must also be provided in the manuscript.

## ChIP-seq

### Data deposition

☒ Confirm that both raw and final processed data have been deposited in a public database such as GEO.

☒ Confirm that you have deposited or provided access to graph files (e.g. BED files) for the called peaks.

| | |
|---|---|
| Data access links<br>*May remain private before publication.* | https://www.ncbi.nlm.nih.gov/geo/query/acc.cgi?acc=GSE131381 |
| Files in database submission | Processed data (.tdf), raw data (bam, bam.bai) and metadate (excel) |
| Genome browser session<br>(e.g. UCSC) | No longer applicable |

### Methodology

| | |
|---|---|
| Replicates | Two technical replicates were used for case (R1881) and control (DMSO) samples |
| Sequencing depth | Libraries were sequenced using an Illumina Hiseq2500 genome analyzer (65bp, single end). Sequencing depth was 10 million reads. |
| Antibodies | Anti-AR (Millipore, 06-680) and anti-H3K27ac (Active Motif, 39133) |
| Peak calling parameters | Peak calling over input control was performed using DFilter (v1.5)37 and MACS for AR and H3k27ac ChIP-seq samples. MACS 1.4 was run with p-value cutoff of 10e-7 and Dfilter with bs=50, ks=30, refine, nonzero. Normalized strand coefficient (NSC) and relative strand correlation (RSC) were calculated using phantompeaktools. |
| Data quality | For ChIP-qPCR enrichment: more than 100 peaks called and NSC, RSC values higher than input samples. |
| Software | DFilter (v1.5), MACS 1.4, ChromHMM (v1.12), Galaxy Cistrome SeqPos motif tool, DiffBind R package (v2.4.6), BEDTools (v2.25). |

## Flow Cytometry

### Plots

Confirm that:

☒ The axis labels state the marker and fluorochrome used (e.g. CD4-FITC).

☒ The axis scales are clearly visible. Include numbers along axes only for bottom left plot of group (a 'group' is an analysis of identical markers).

☒ All plots are contour plots with outliers or pseudocolor plots.

☒ A numerical value for number of cells or percentage (with statistics) is provided.

### Methodology

| | |
|---|---|
| Sample preparation | Freshly collected biopsies were chopped in PBS and processed using a gentleMACS Dissociator (Miltenyi Biotec). Programs used for human tissue were "h_tumour_01" for one time and "h_tumour_03" for three times. The cell suspension was subsequently filtered using a 70µm nylon cell strainer (BD Biosciences) and washed with PBS. Cells were centrifuged at 1200 rpm for 6 min and resuspended in PBS. Cell suspension was stained for flow cytometry sorting with CD14-PE and CD11b-PE antibody (eBioscience) in PBS + 0.5% BSA for 20 min at 4°C |
| Instrument | LSR Fortessa SORP1 flow cytometer and MoFlo Astrios Beckman Coulter |
| Software | FlowJo V9 |

| | |
|---|---|
| Cell population abundance | Abundance of target cells was <1% |
| Gating strategy | Macrophage like cells were first selected based on FSC-SSC, followed by doublets exclusion and DAPI-negative selection. CD45- cells were then excluded. CD45+ cells with low SCC scatter were excluded as possible T lymphocytes. CD14+ and/or CD11b+ macrophages were isolated in 384 well plates. |

☒ Tick this box to confirm that a figure exemplifying the gating strategy is provided in the Supplementary Information.
