# Supplementary material for: Androgen receptor signalling in macrophages promotes TREM-1-mediated prostate cancer cell line migration and invasion
Source: Nat Commun. 2020 Sep 9;11:4498. doi: 10.1038/s41467-020-18313-y (PMC7481219; doi:10.1038/s41467-020-18313-y)
Supplement: Supplementary file 4 — Source Data [file 41467_2020_18313_MOESM4_ESM.zip › Source data western blots and scratch assays.pptx]

## Slide 1
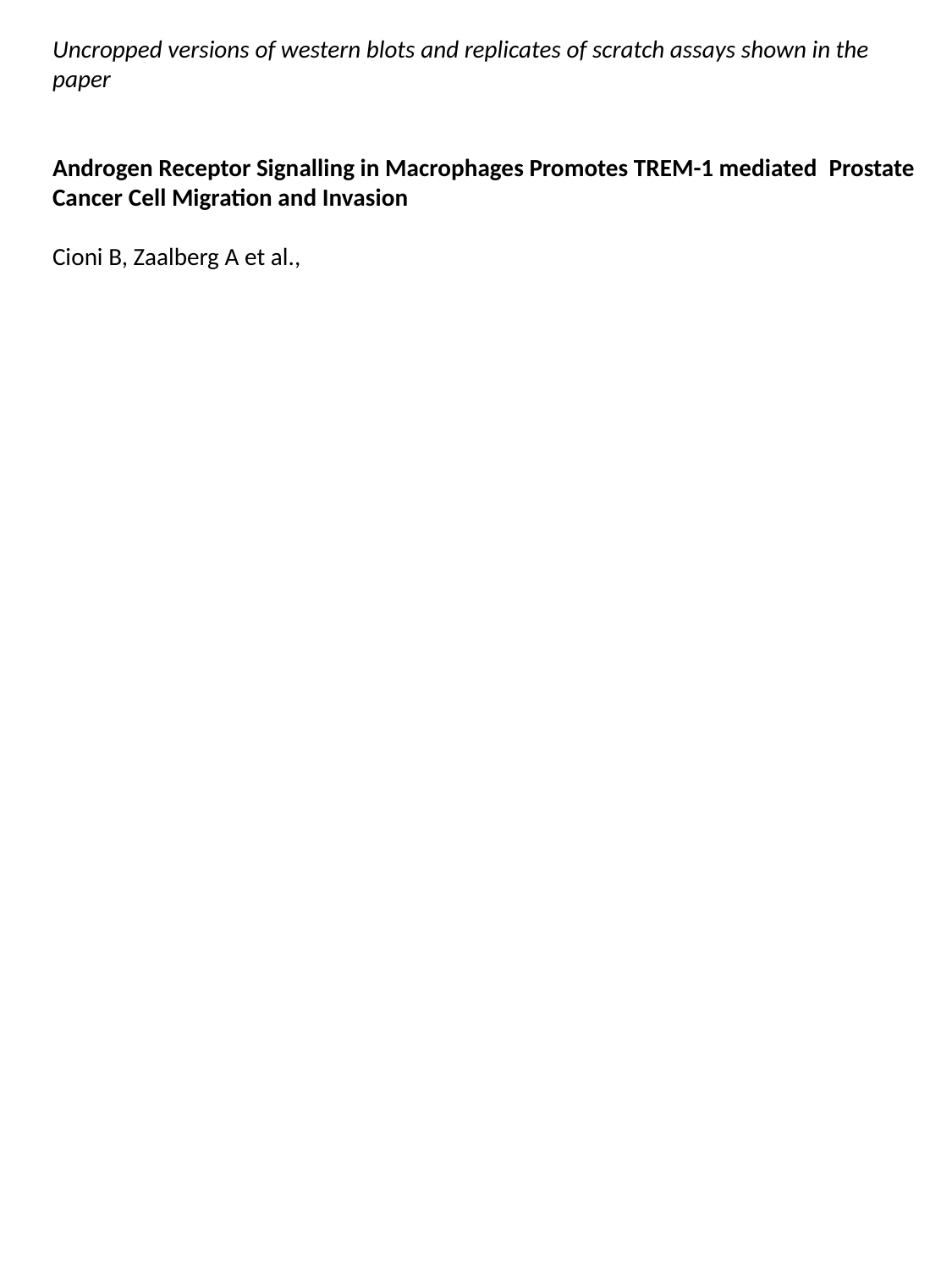

Uncropped versions of western blots and replicates of scratch assays shown in the paper
Androgen Receptor Signalling in Macrophages Promotes TREM-1 mediated  Prostate Cancer Cell Migration and Invasion
Cioni B, Zaalberg A et al.,

## Slide 2
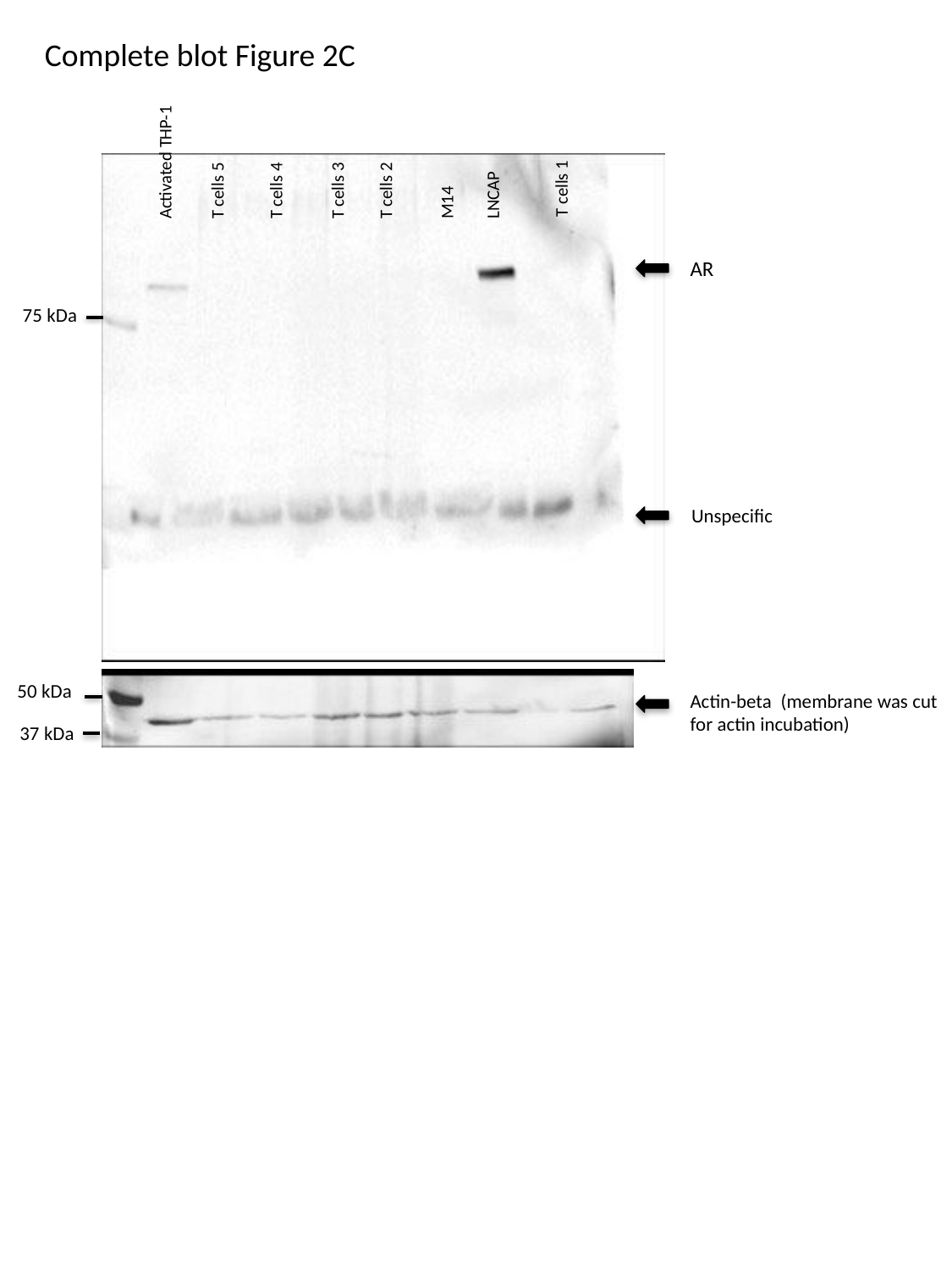

Complete blot Figure 2C
Activated THP-1
T cells 1
T cells 2
T cells 3
T cells 4
T cells 5
LNCAP
M14
AR
75 kDa
Unspecific
50 kDa
Actin-beta (membrane was cut
for actin incubation)
37 kDa

## Slide 3
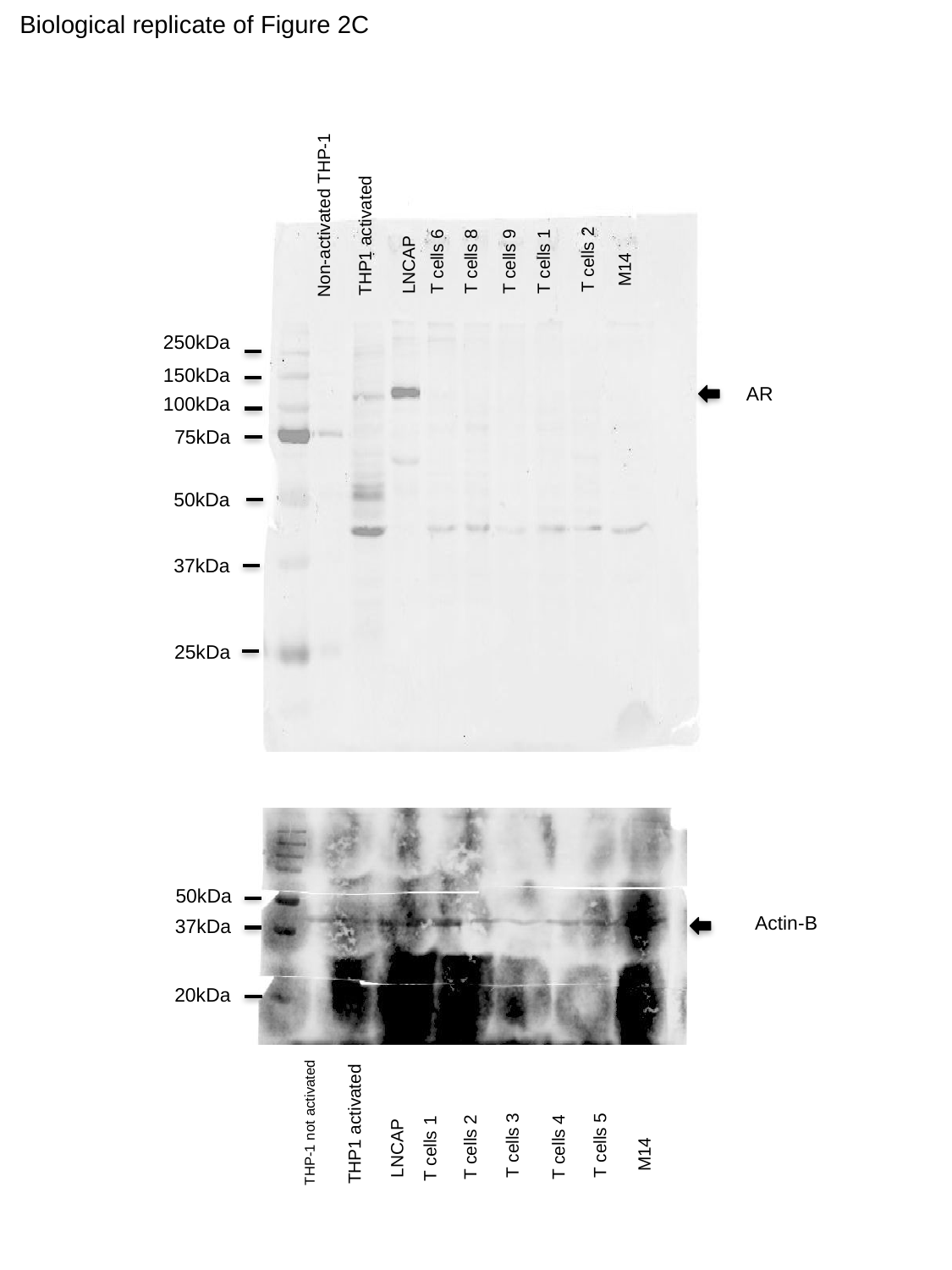

Biological replicate of Figure 2C
50kDa
Actin-B
20kDa
Non-activated THP-1
THP1 activated
T cells 2
T cells 8
T cells 1
T cells 6
T cells 9
LNCAP
M14
AR
75kDa
THP1 activated
THP-1 not activated
T cells 3
T cells 5
T cells 2
T cells 4
LNCAP
T cells 1
M14
250kDa
150kDa
100kDa
50kDa
37kDa
25kDa
37kDa

## Slide 4
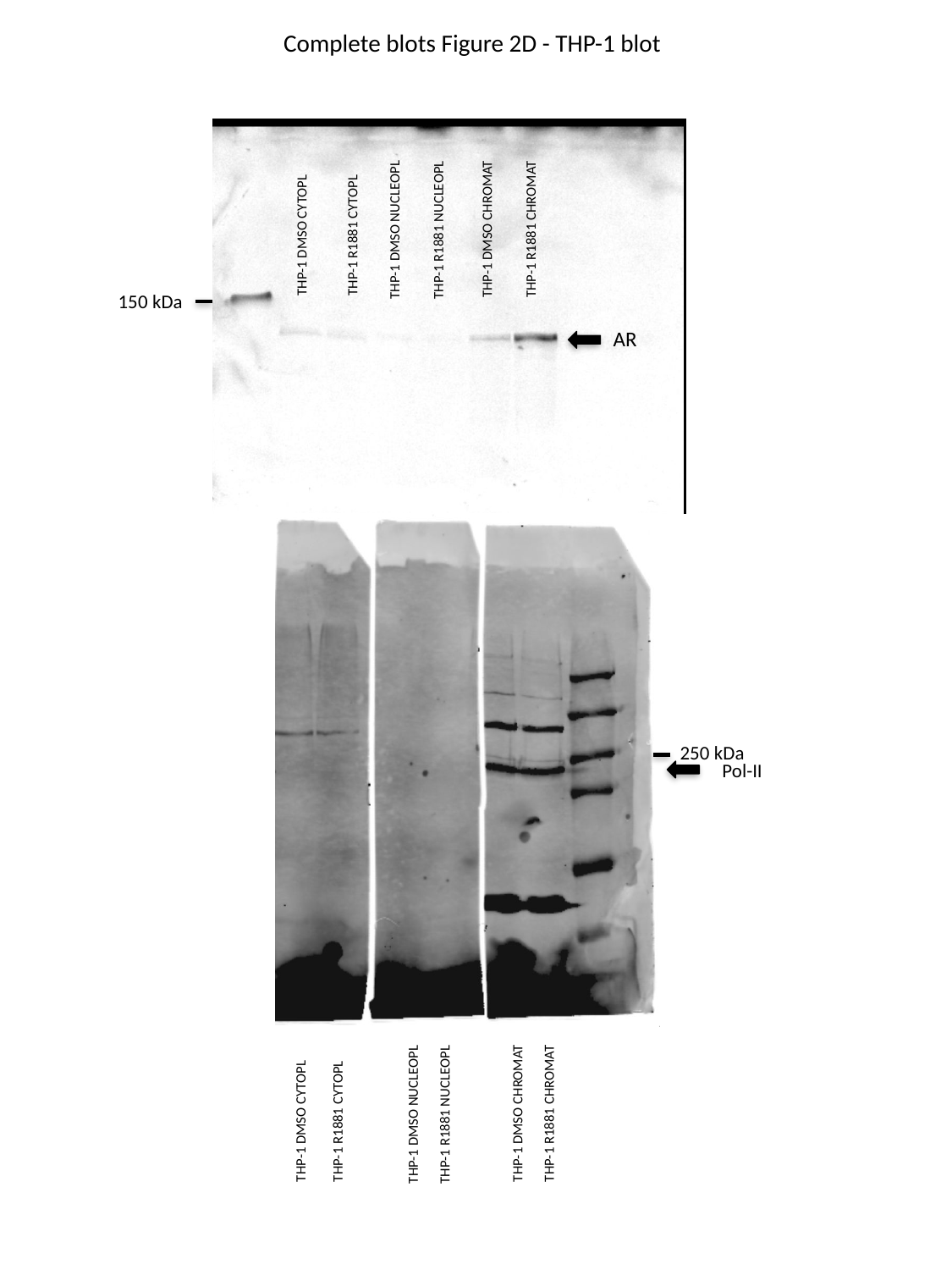

Complete blots Figure 2D - THP-1 blot
THP-1 R1881 CYTOPL
THP-1 DMSO CYTOPL
THP-1 R1881 CHROMAT
THP-1 DMSO CHROMAT
THP-1 R1881 NUCLEOPL
THP-1 DMSO NUCLEOPL
150 kDa
AR
THP-1 R1881 CYTOPL
THP-1 DMSO CYTOPL
THP-1 R1881 CHROMAT
THP-1 DMSO CHROMAT
THP-1 R1881 NUCLEOPL
THP-1 DMSO NUCLEOPL
250 kDa
Pol-II

## Slide 5
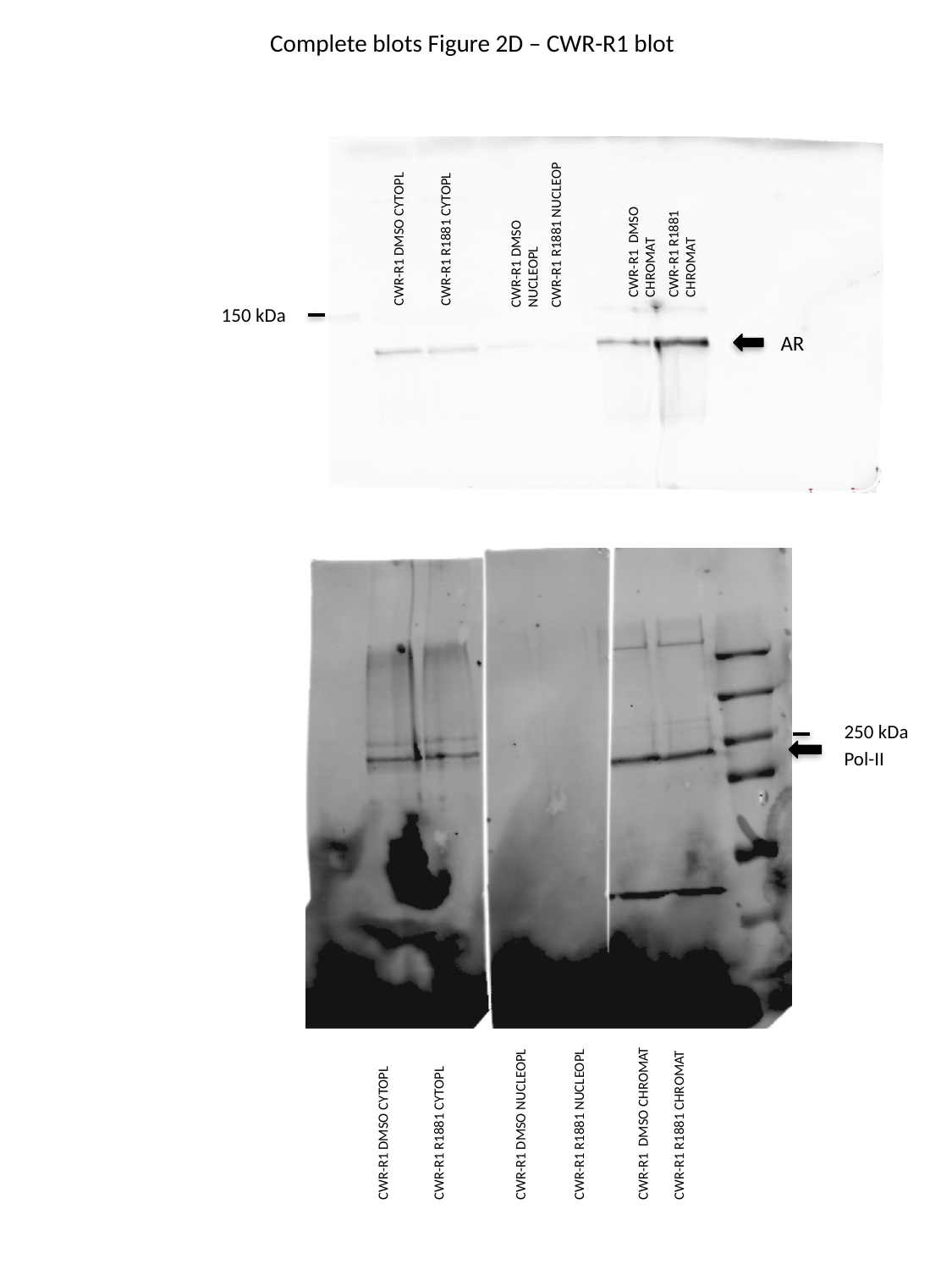

Complete blots Figure 2D – CWR-R1 blot
CWR-R1 R1881 CHROMAT
CWR-R1 DMSO CHROMAT
CWR-R1 R1881 CYTOPL
CWR-R1 DMSO CYTOPL
CWR-R1 R1881 NUCLEOP
CWR-R1 DMSO NUCLEOPL
150 kDa
AR
CWR-R1 R1881 CHROMAT
CWR-R1 DMSO NUCLEOPL
CWR-R1 R1881 CYTOPL
CWR-R1 DMSO CYTOPL
CWR-R1 DMSO CHROMAT
CWR-R1 R1881 NUCLEOPL
250 kDa
Pol-II

## Slide 6
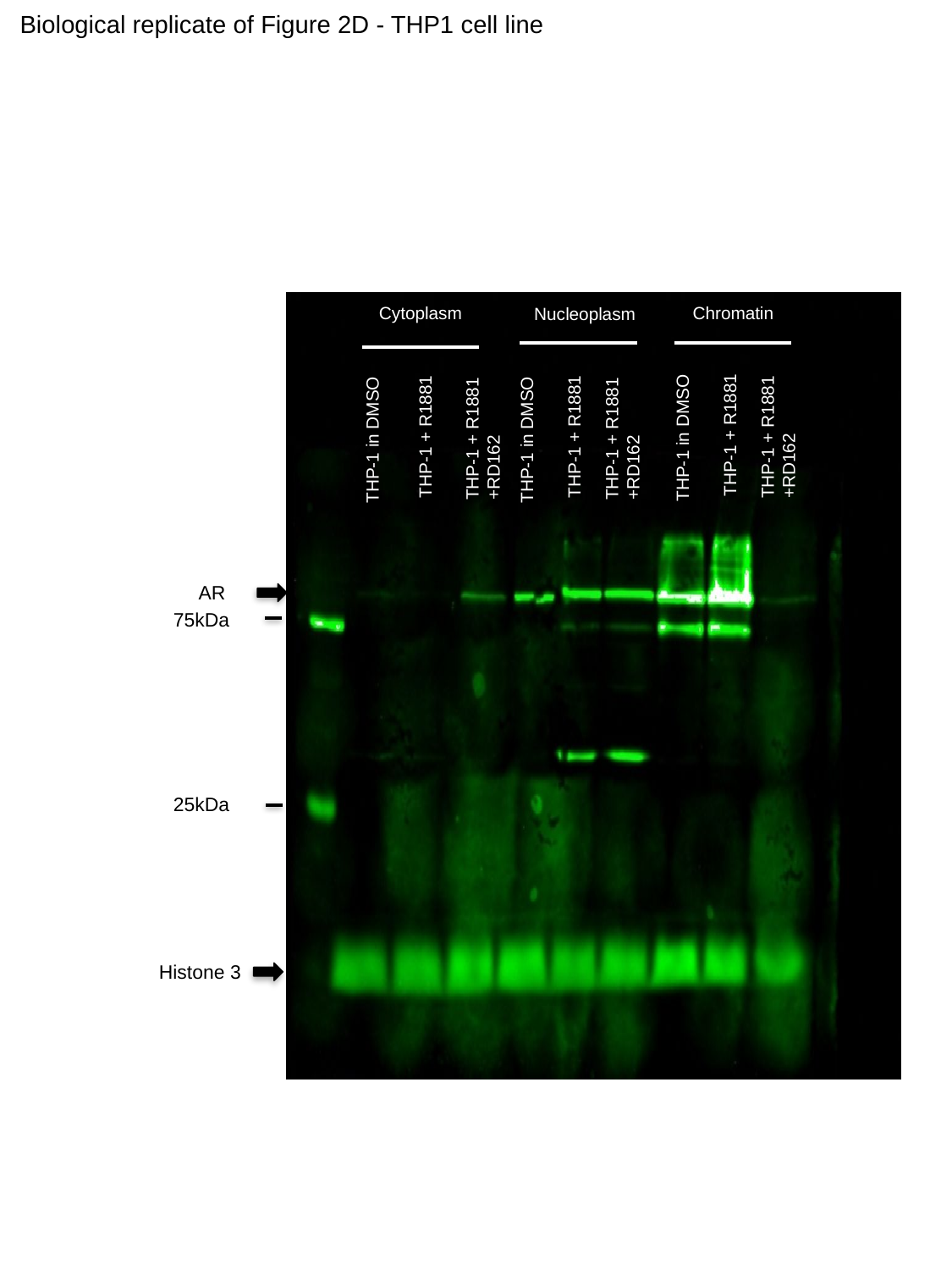

Biological replicate of Figure 2D - THP1 cell line
Cytoplasm
Chromatin
Nucleoplasm
THP-1 + R1881
+RD162
THP-1 + R1881
+RD162
THP-1 + R1881
+RD162
THP-1 + R1881
THP-1 + R1881
THP-1 + R1881
THP-1 in DMSO
THP-1 in DMSO
THP-1 in DMSO
AR
75kDa
25kDa
Histone 3

## Slide 7
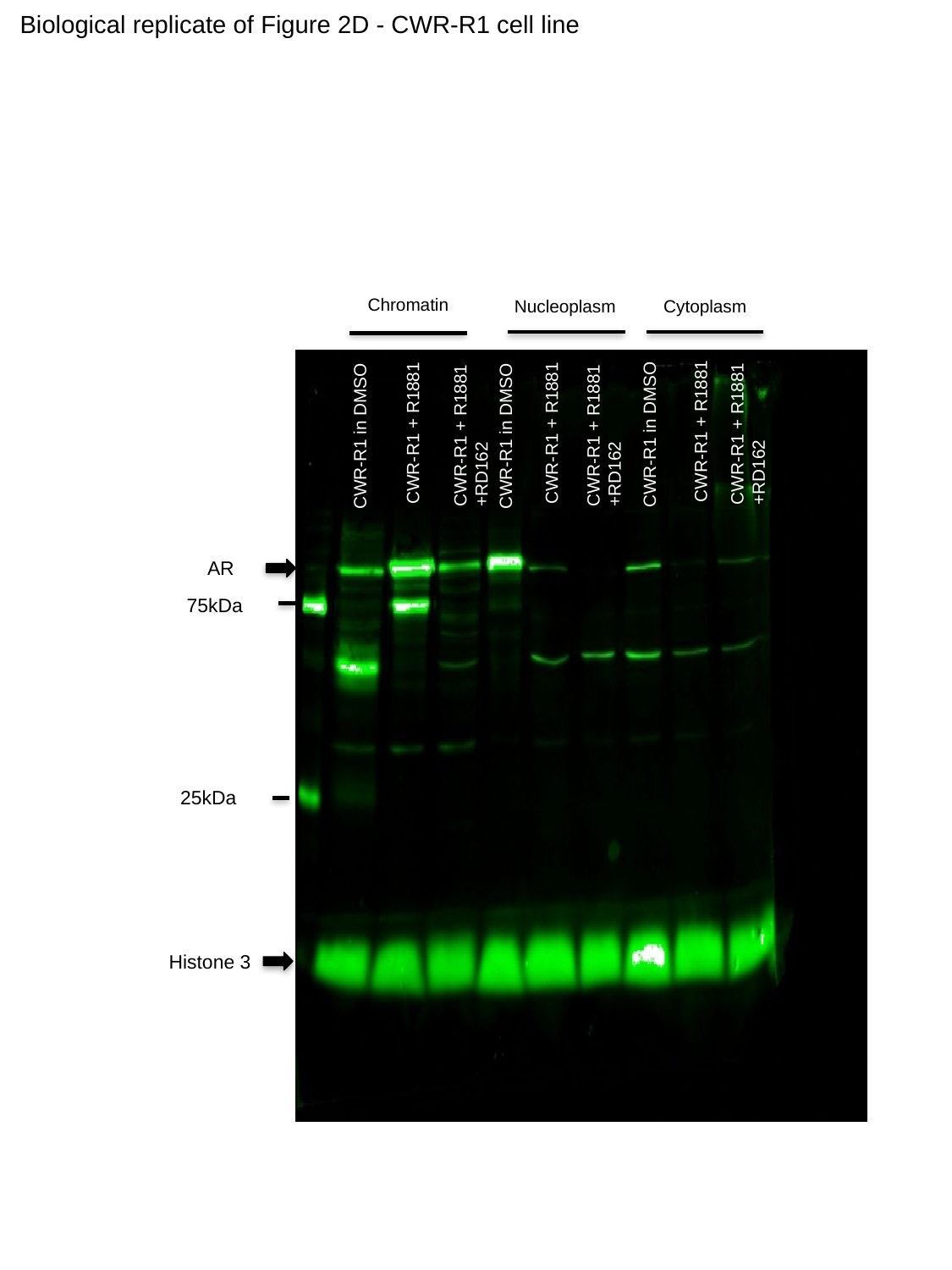

Biological replicate of Figure 2D - CWR-R1 cell line
Chromatin
Nucleoplasm
Cytoplasm
CWR-R1 + R1881
+RD162
CWR-R1 + R1881
+RD162
CWR-R1 + R1881
+RD162
CWR-R1 + R1881
CWR-R1 + R1881
CWR-R1 + R1881
CWR-R1 in DMSO
CWR-R1 in DMSO
CWR-R1 in DMSO
AR
75kDa
25kDa
Histone 3

## Slide 8
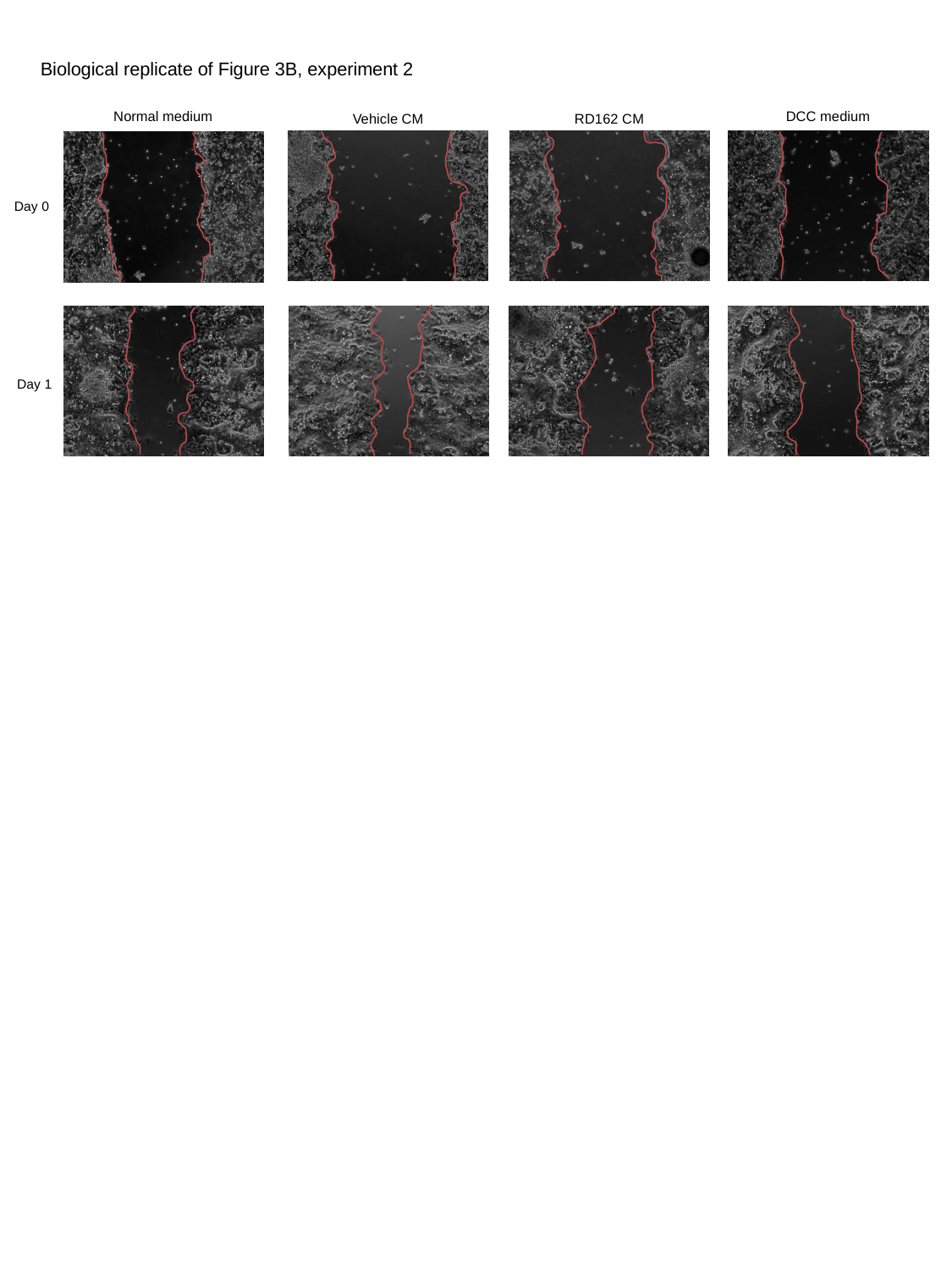

Biological replicate of Figure 3B, experiment 2
DCC medium
Normal medium
Vehicle CM
RD162 CM
Day 0
Day 1

## Slide 9
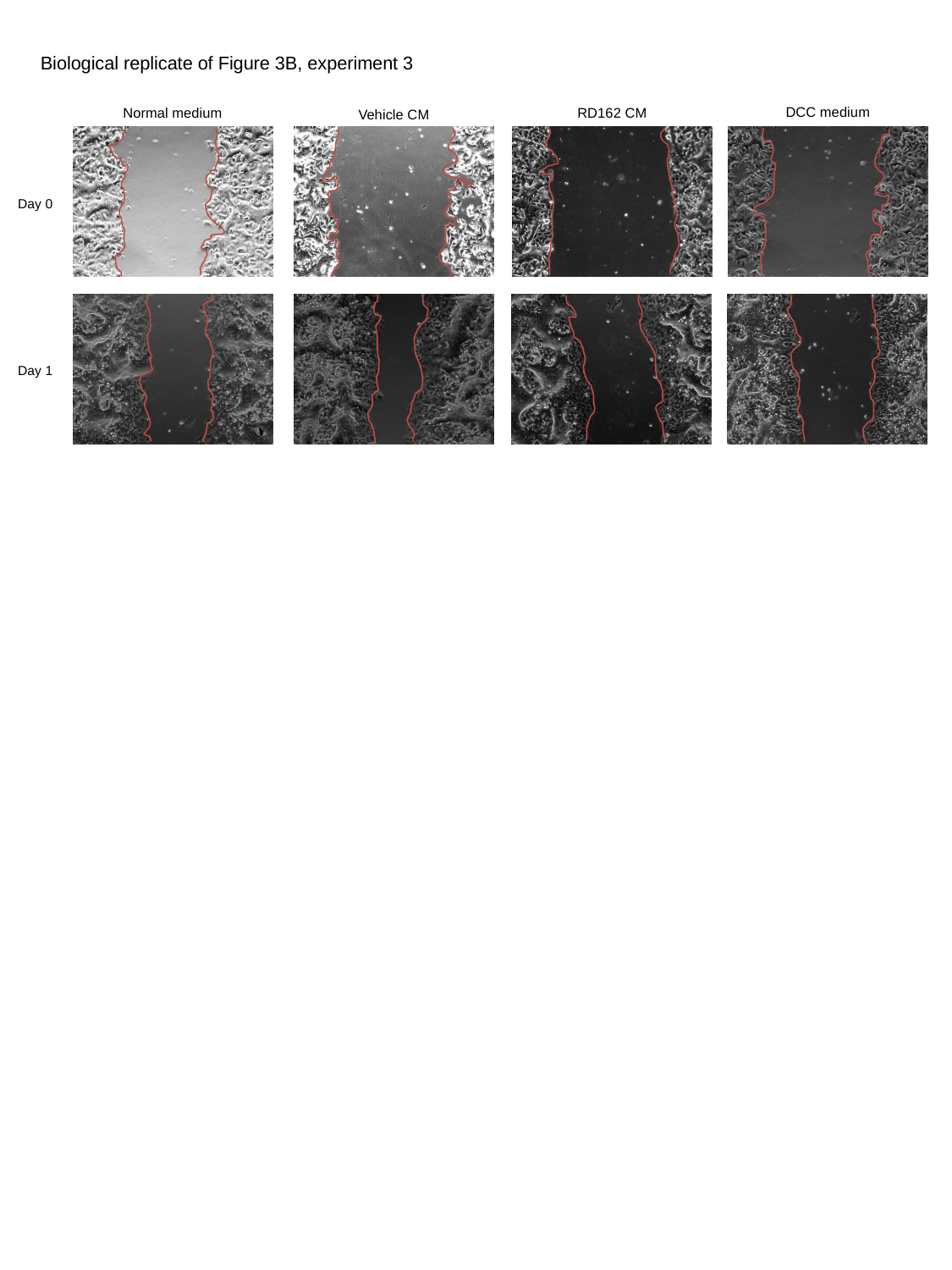

Biological replicate of Figure 3B, experiment 3
DCC medium
RD162 CM
Normal medium
Vehicle CM
Day 0
Day 1

## Slide 10
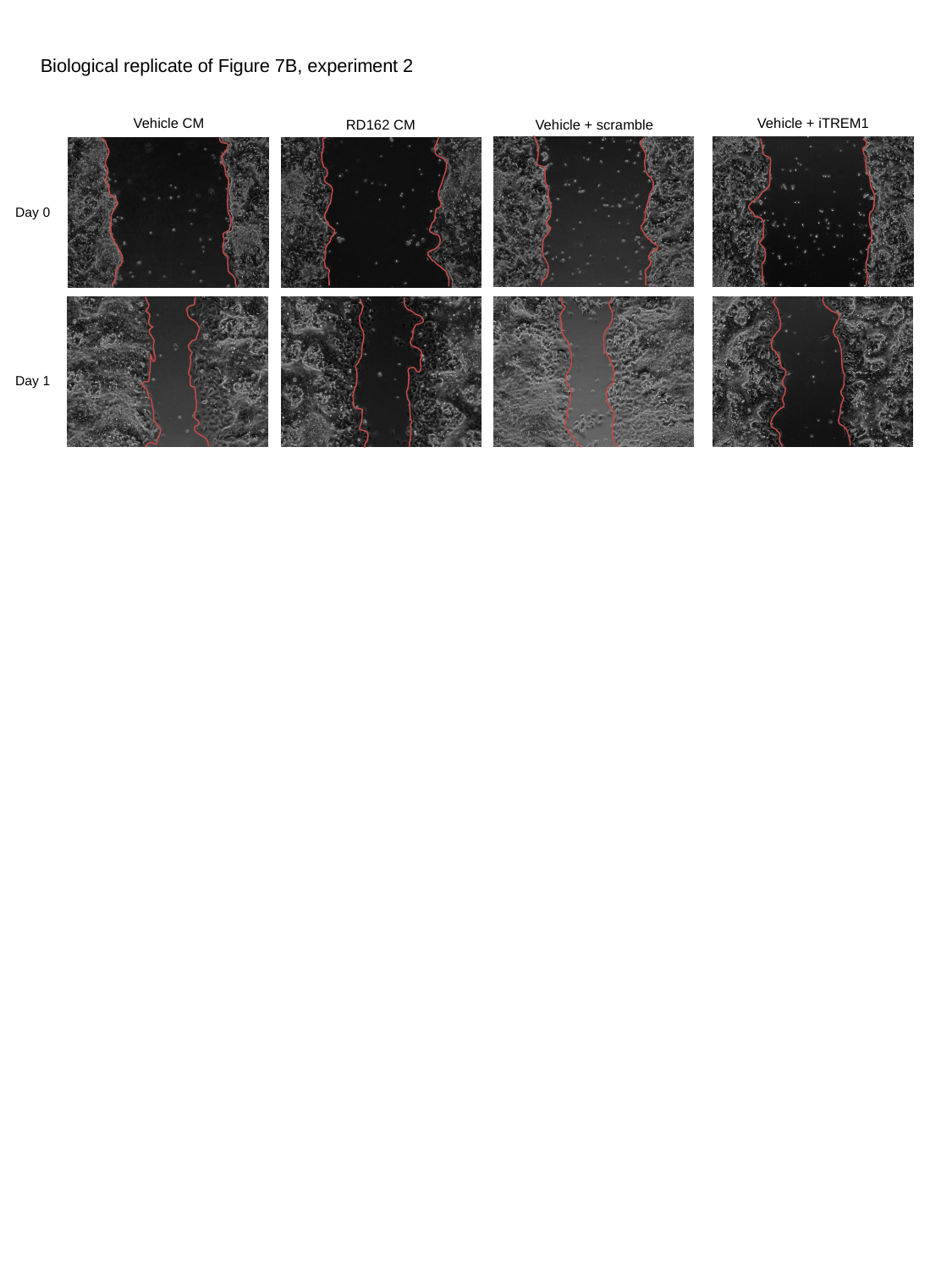

Biological replicate of Figure 7B, experiment 2
Vehicle + iTREM1
Vehicle CM
RD162 CM
Vehicle + scramble
Day 0
Day 1

## Slide 11
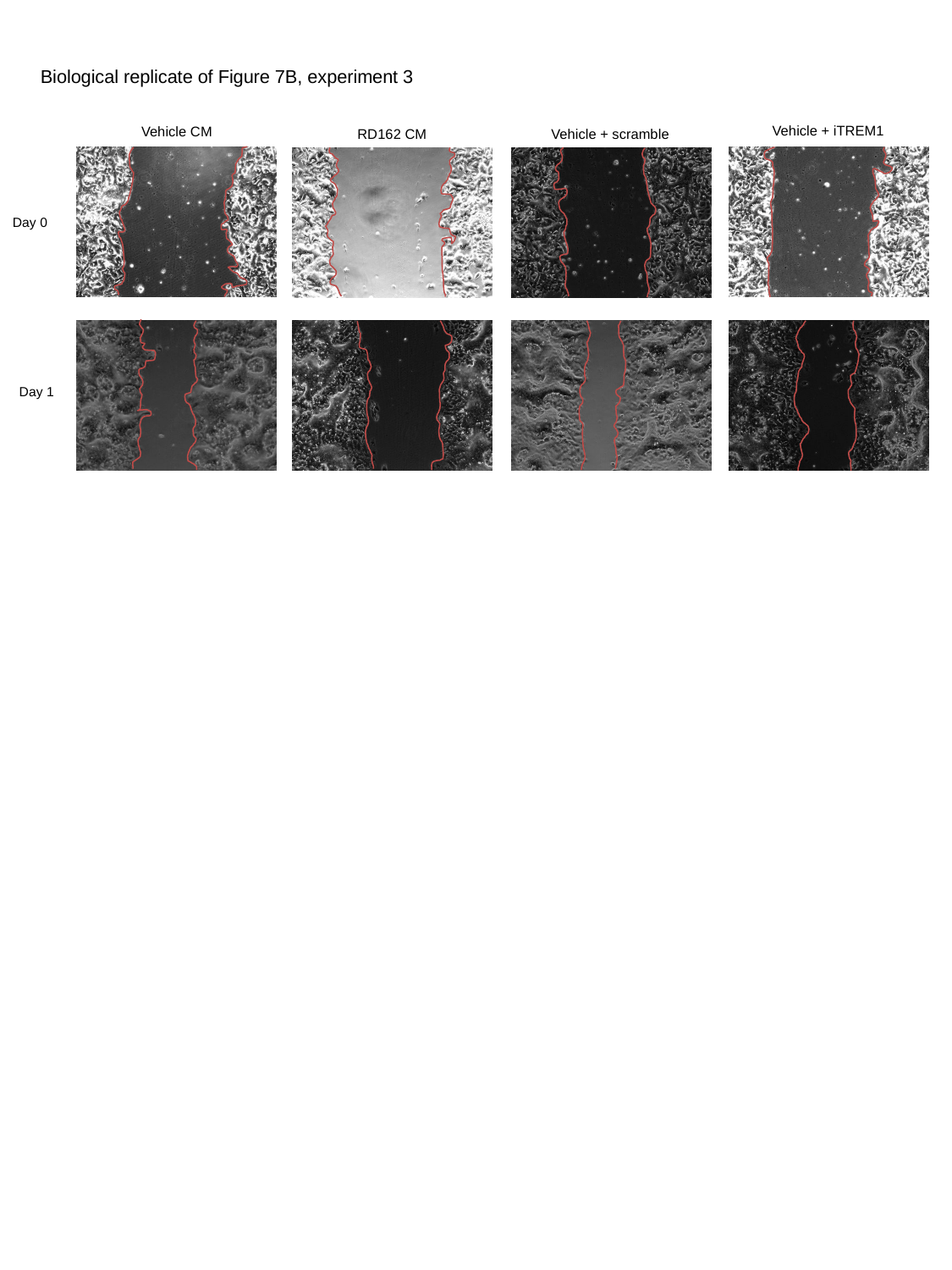

Biological replicate of Figure 7B, experiment 3
Vehicle + iTREM1
Vehicle CM
RD162 CM
Vehicle + scramble
Day 0
Day 1

## Slide 12
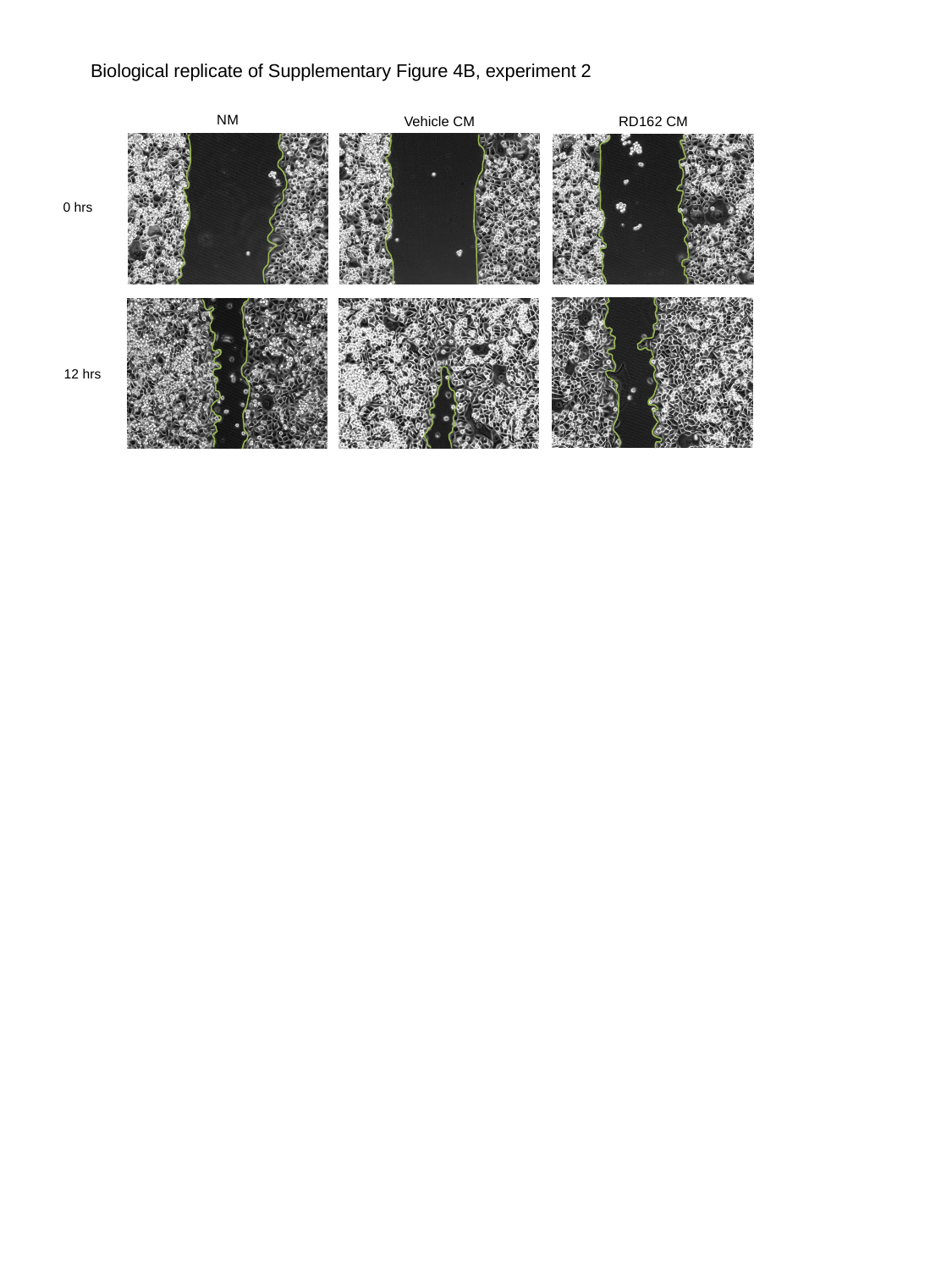

Biological replicate of Supplementary Figure 4B, experiment 2
NM
Vehicle CM
RD162 CM
0 hrs
12 hrs
